# Knockout of mitochondrial dicarboxylate carriers AtDIC1 and AtDIC3 affects germination, biomass accumulation, and carbon partitioning in *Arabidopsis thaliana*

**DOI:** 10.1007/s00299-026-04000-7

**Published:** 2026-09-28

**Authors:** Mariana de Lara Campos Arcuri, Pedro Barreto, Andressa Nagatani Marinho, Luís Paulo Benetti Mantoan, Ilara Gabriela Frasson Budzinski, Felipe Girotto Campos, Ivan G. Maia

**Affiliations:** 1 Department of Genetics, Microbiology and Immunology, Institute of Biosciences, São Paulo State University (UNESP), Botucatu, SP Brazil; 2 Plant Energy Biology, Institute of Plant Biology and Biotechnology, University of Münster, Münster, Germany; 3 Department of Genetics, Max Feffer Laboratory of Plant Genetics, College of Agriculture Luiz de Queiroz, University of São Paulo, Piracicaba, SP Brazil; 4 Department of Biodiversity and Biostatistics, Institute of Biosciences, São Paulo State University (UNESP), Botucatu, SP Brazil

**Keywords:** Mitochondrial Dicarboxylate Carrier (DIC), Mitochondrial Carrier Family, Organic acid transport, Plant mitochondria, T-DNA insertion mutants

## Abstract

**Key message:**

AtDIC1 and AtDIC3 make distinct, partially overlapping contributions to mitochondrial metabolism, influencing germination performance, carbon allocation, and redox balance in *Arabidopsis thaliana*.

**Abstract:**

The mitochondrial dicarboxylate carrier (DIC) is essential for the exchange of dicarboxylic acids across the inner mitochondrial membrane. Although three DIC homologues (*AtDIC1–3*) are present in *Arabidopsis thaliana*, the functional roles of AtDIC1 and AtDIC3 remain poorly understood. In this study, we characterized *atdic1* and *atdic3* single and double mutants to elucidate their physiological significance. Loss of either isoform delayed seed germination under both control and abiotic stress conditions. This delay was more pronounced in *atdic1*, particularly on sucrose-free medium, suggesting a requirement for AtDIC1 in the mobilization of endogenous carbon reserves. During vegetative development, the *atdic3* mutant showed reduced primary root elongation under salt and osmotic stress, whereas *atdic1* displayed normal or enhanced root growth under salinity, revealing distinct stress-response roles. Notably, all mutant lines exhibited increased shoot biomass compared to the wild type, supported by enhanced photosynthetic efficiency, improved chlorophyll re-oxidation, and a shift in carbon allocation toward starch at the expense of sucrose. Double mutant analysis showed no additive effects, suggesting that these isoforms do not act synergistically. Metabolomic and redox profiling demonstrated that *atdic3* disruption specifically decreased the NADH/NAD+ ratio and increased NADPH/NADP+ levels, coupled with amino acid accumulation. Conversely, *atdic1* mutants showed more substantial disruptions in sugar and ascorbate metabolism. Although *AtDIC2* was upregulated in all mutant backgrounds, it provided only partial functional compensation. Our findings indicate that AtDIC1 and AtDIC3 fulfill distinct yet partially overlapping roles in coordinating mitochondrial metabolism with plant growth and environmental adaptation.

**Supplementary Information:**

The online version contains supplementary material available at https://doi.org/10.1007/s00299-026-04000-7.

## Introduction

Dicarboxylates are essential components of various metabolic pathways in plants, acting as intermediates and precursors in processes critical for plant growth, development, and survival (Wiskich 1977). Beyond their involvement in fatty acid metabolism, amino acid synthesis, and gluconeogenesis, dicarboxylates are also implicated in photorespiration, particularly through the transfer of reducing equivalents (Wiskich 1977; Palmieri et al. 2008). Different members of the mitochondrial carrier family (SLC25), including the Mitochondrial Dicarboxylate Carrier (DIC), can mediate the transport of dicarboxylic acids across the inner mitochondrial membrane (Palmieri 2013; Fernie et al. 2020). In the model plant *Arabidopsis thaliana,* three DIC homologues with distinct substrate specificities, compared to their yeast and animal counterparts, have been identified (Palmieri et al. 2008). AtDIC1 and AtDIC2 share 70% of identical amino acid residues and are presumed to be ubiquitously expressed. In contrast, AtDIC3, which shares 55–60% aa identity with the other isoforms, is considered an early genome acquisition exclusively found in Brassicaceae and displays a more restricted expression pattern (Palmieri et al. 2008; Barreto et al. 2022).

In proteoliposomes, recombinant DIC proteins can exchange sulfate with malate, oxaloacetate, phosphate, and succinate, as well as, to a lesser extent, with 2-oxoglutarate (Palmieri et al. 2008). In vivo, however, AtDIC2 has been shown to facilitate the export of cytosolic malate in exchange for mitochondrial citrate, a crucial function for maintaining metabolic homeostasis, especially under stress conditions (Lee et al. 2021). Consequently, AtDIC2 is proposed to play a pivotal role in regulating anaplerotic metabolism, effectively linking assimilatory and catabolic pathways between mitochondria and other cellular compartments (Lee et al. 2021). The knockout of *AtDIC2* in *A. thaliana* led to impaired vegetative growth. Notably, despite the high amino acid similarity among the three isoforms, neither AtDIC1 nor AtDIC3 could functionally compensate for the loss of AtDIC2 (Lee et al. 2021). This finding indicates that these isoforms have distinct, non-redundant functions critical to their specific physiological roles in plants.

Gene ontology enrichment analysis indicates roles for *AtDIC1* and *AtDIC2* in general stress responses, whereas *AtDIC3* appears to be involved in phosphate starvation (Nunes-Nesi et al. 2020; Barreto et al. 2022). In line with this, environmental stressors have been shown to markedly upregulate the expression of *AtDIC1* and *AtDIC2* (Van Aken et al. 2009). Moreover, all three Arabidopsis DICs exhibit strong upregulation in response to touch-induced mechanical stimulation (Lee et al. 2005; Van Aken et al. 2016), a response considered an integral part of intrinsic physiological adjustments. Interestingly, overexpression of *AtDIC2* in Arabidopsis provided evidence of its involvement in the adaptive response to hypoxia (Barreto et al. 2022). Hypoxia significantly impacts the tricarboxylic acid (TCA) cycle, leading to altered levels of its intermediates, most notably the accumulation of succinate and malate (van Veen et al. 2025). Given their role in transporting these metabolites, DICs may contribute to the metabolic adjustments required for plant adaptation to low-oxygen conditions. Collectively, these findings suggest that modulation of DIC activity and expression constitutes a key component of the metabolic reprogramming strategy employed by plants to cope with adverse growth conditions.

In contrast to the functional data available for AtDIC2, the physiological roles of AtDIC1 and AtDIC3 remain largely unexplored. In this study, by employing single and double insertion mutants of *AtDIC1* and *AtDIC3*, we demonstrate that the reduced expression of these genes leads to delayed germination and diminished seed yield. Intriguingly, we observed an unexpected increase in leaf area and plant biomass, indicative of enhanced vegetative growth of the mutant lines. These phenotypic alterations were accompanied by improved photosynthetic capacity, altered levels of carbohydrates, organic acids, and amino acids, and a concomitant upregulation of *AtDIC2* expression.

## Materials and methods

### Plant material and growth conditions

Single *A. thaliana* mutants carrying T-DNA insertions in the *AtDIC1* (At2g22500; SALK_090789 and SALK_067118) and *AtDIC3* (At5g09470; SALK_001547 and SALK_087822C) genes were obtained from the Arabidopsis Biological Resource Center. All mutants were in the Columbia (Col-0) ecotype background. Plants were grown in plastic pots containing a soil-vermiculite mixture (2:1) and maintained in a growth chamber (Model CPM6050, Conviron, Pembina, ND, USA) under a 16-h light/8-h dark photoperiod, with day/night temperatures of 21/18 ± 1 °C and a light intensity of 100 µE m^−2^ s^−1^. Samples from various organs/tissues at different developmental stages were collected and immediately frozen in liquid nitrogen for subsequent RNA extraction and relative expression analysis via RT-qPCR. The sampled organs/tissues included germinating seeds, 14-day-old seedlings, vegetative rosette leaves (growth stage 3.5; Boyes et al. 2001), stems, flowers at stages 9 and 15 (as classified by Smyth et al. 1990), and siliques.

For the germination assays, wild type (WT) and mutant seeds (n=30/genotype/dish) were surface-sterilized and sown on Petri dishes containing half-strength Murashige and Skoog medium (1/2 MS; Murashige & Skoog 1962) plus Phytagel (0.8%) and sucrose (30 g·L^−1^), supplemented or not with 100 mM NaCl or 200 mM mannitol (Arcuri et al. 2021). A second assay was performed to evaluate the influence of a carbon source on germination by using the same 1/2 MS-Phytagel dishes without sucrose. After 4 days of stratification (DAS) at 4 °C in the dark, the dishes were transferred to the growth chamber, and seed germination (determined by radicle protrusion) was scored daily for 4 to 8 days. Three independent experiments, each with a separate seed lot, were conducted. Germination assays for gene expression analysis were conducted using three biological replicates essentially as described (Matiolli et al. 2011). Briefly, seeds (50 mg) from WT Col-0 and mutant lines were surface-sterilized and incubated in 50 ml of 1/2 MS medium, with or without sucrose (30 g·L^−1^), for 3 days at 4 °C in the dark to break dormancy. Afterward, the germinating seeds were kept under a long-day photoperiod with constant agitation (67 rpm) and collected at different time points (1, 2, 3 and 4 DAS) for total RNA extraction and subsequent RT-qPCR analysis.

For primary root elongation measurements, 10-day-old seedlings (n = 5/genotype) were grown vertically on Petri dishes (three per treatment) containing 1/2 MS–0.8% Phytagel medium supplemented or not with 100 mM NaCl or 200 mM mannitol (Arcuri et al. 2021). After 10 days under the described growth conditions, digital images were captured, and the primary root length was determined using ImageJ software.

### Molecular characterization of *atdic* mutants and phenotyping

Homozygous lines for each mutant were screened via genomic PCR using a combination of *AtDIC1*- or *AtDIC3*-specific primers and the T-DNA left border primer (SALK LBb1.3) (Primers listed in Supplementary Table S1) essentially as described (Arcuri et al. 2021). The identity of the mutated genes and T-DNA insertion sites was confirmed by sequencing the PCR products encompassing the T-DNA flanking regions. During phenotypic analysis, homozygous mutant lines and WT plants (n=15/genotype) were photographed prior to flowering, and the following parameters were evaluated: rosette area over time, fresh and dry weights of the aerial parts, time to flowering, silique length, and seed yield. Rosette area was determined using the ImageJ software. To calculate average silique length, 10 siliques per plant were measured. Seed yield was determined by weighing the seeds harvested from 60 siliques per plant (n=3 plants/genotype). Average seed weight was determined by weighing 100 mature dry seeds using a microbalance, with three biological replicates (independent seed lots). To generate the double mutant *atdic1/atdic3*, crosses were performed between the homozygous single lines with the greatest reductions in *AtDIC1* and *AtDIC3* transcript levels (specifically, *atdic1-1* and *atdic3-1*). Double mutant homozygous plants in the F2 generation were confirmed via PCR and target gene expression verified using RT-qPCR.

### RNA extraction, cDNA preparation and expression analysis

Total RNA was extracted using TRIzol reagent (Invitrogen, Waltham, MA, USA) following the manufacturer’s instructions. RNA integrity was assessed via agarose–formaldehyde gel electrophoresis, and its concentration was measured using a UV spectrophotometer. To remove genomic DNA contamination, the extracted RNA was treated with RQ1 RNase-Free DNase (Promega, Madison, WI, USA) before complementary DNA (cDNA) synthesis. First-strand cDNA was synthesized from 1 μg of total RNA using the High-Capacity cDNA Reverse Transcription Kit (Thermo Fisher Scientific, Waltham, MA, USA) according to the manufacturer’s protocol. Samples were then treated with RNase A (Thermo Fisher Scientific). Transcript levels of target genes were quantified using quantitative PCR (qPCR) on a StepOnePlus Real-Time PCR System (Applied Biosystems, Waltham, MA, USA). The cycling parameters were as follows: initial denaturation at 95 °C for 10 min, followed by 40 cycles of 94 °C for 15 s, 60 °C for 30 s, and an extension at 72 °C for 30 s. Each 10 µl reaction contained 5 µl of GoTaq Colorless qPCR Master Mix (Promega, Madison, WI, USA), 1 µl of cDNA (1:10 dilution), and 0.3 µM each of forward and reverse primers (Supplementary Table S1). All reactions were performed in triplicate. Relative expression levels were calculated using the 2^−ΔΔCt^ method (Livak and Schmittgen 2001) and analyzed with DataAssist software v3.01 (Applied Biosystems). *GAPDH* and *40S ribosomal protein S16* (Czechowski et al. 2005) were used as reference genes for normalization (Supplementary Table S1).

### Measurements of gas-exchange and chlorophyll *a* fluorescence

Gas-exchange parameters were measured in entire plants (35 DAS) from WT and single mutant lines (n = 6/genotype), simultaneously with chlorophyll *a* fluorescence measurement, using an infrared gas analyzer (GSF 3000, Walz, GmbH, Effeltrich, Germany) with air flow of 300 mL min^−1^ and 1200 µmol m^−2^∙s^−1^ coupled light source. All measurements were performed between 9 and 10 o’clock (3 h after lights on) under the following conditions: ambient CO_2_ concentration of 398 ± 6 μmol mol^−1^, temperature of 26 ± 2 °C and 58% relative humidity. The following parameters were measured: net photosynthetic rate (*A*_*n*_; µmol m^−2^∙s^−1^), stomatal conductance (*gs*; mmol m^−2^∙s^−1^) and transpiration rate (*E*; mmol m^−2^∙s^−1^). Chlorophyll *a* fluorescence was recorded with a coupled modulated light fluorometer (LED-Array/PAM-Fluorometer 3055-FL, Walz, GmbH, Effeltrich, Germany). The following parameters were calculated: PSII maximum efficiency in the light-adapted state (*Fv′/Fm′*), the effective quantum efficiency of PSII [Y(II) or ΦPSII = (Fm′ − F)/Fm′ = qP × (Fv′/Fm′)], and photochemical quenching [qP = (Fm′ − F)/(Fm′ − Fo′)]. Chlorophyll *a* fluorescence light-response curves were measured using a pulse-amplitude-modulated fluorometer (JUNIOR-PAM, Walz, Germany) under eight increasing photosynthetic photon flux densities (PPFD: 125, 190, 285, 420, 625, 845, 1150, and 1500 µmol photons m⁻^2^∙s⁻^1^). Three biological replicates were analyzed per genotype. Measurements were performed at the end of the dark period, prior to the onset of the photoperiod. Minimal fluorescence of dark-adapted leaves (F₀) was recorded, followed by a 0.6-s saturating pulse (10,000 µmol photons m⁻^2^∙s⁻^1^) to determine maximal fluorescence (Fm). Each PPFD level was then applied for 40 s to reach steady-state fluorescence (Fs), after which a saturating pulse was used to determine maximal fluorescence in the light-adapted state (Fm′), and a brief far-red pulse to obtain minimal fluorescence (Fo′). This procedure was repeated sequentially for all PPFD levels.

### Metabolic profiling

Metabolites were extracted from whole plant rosettes (35 DAS) harvested at the midpoint of the photoperiod. Frozen tissue (50 mg) was ground in liquid nitrogen and incubated with 1 mL of extraction solution (90% methanol supplemented with 2 mg/mL ribitol and 6 mg/mL adipic acid as internal standards) for 20 min at 75 °C. The samples were then shaken for 15 min. Cellular debris was removed by centrifugation at 12.000 rpm for 5 min at 4 °C. The supernatant was filtered through 0.2 µm filters, and 900 µL of the filtered extract was transferred to 1.5 mL amber glass vials. Samples were dried and derivatized in two steps as described (Budzinski et al. 2019). Metabolite analysis was performed using an Agilent 7890A gas chromatograph (Agilent Technologies, Santa Clara, USA) coupled to a Leco Pegasus BT GC-TOF/MS (Pegasus 4D, Leco Corp., St. Joseph, USA). Statistical analyses of relative metabolite intensities and metabolic deviations were conducted using MetaboAnalyst 8.0 (https://www.metaboanalyst.ca/home.xhtml). For global metabolite analysis, no filters or normalization were applied; data were log-transformed (log_2_) and scaled using Pareto scaling. For pathway analysis of altered metabolic processes, default settings were used. Input metabolites included those identified as differentially abundant in the ANOVA from the prior analysis. Five biological replicates were analyzed per genotype.

### Redox state, quantification of carbohydrates and photosynthetic pigments

Assays were performed using entire rosettes (35-DAS) from WT and mutant lines harvested at the middle of the light period to ensure a representative state of active photosynthetic metabolism and stable carbon partitioning. Redox status was determined by quantifying total NAD⁺, NADH, NADP⁺, and NADPH using the NAD/NADH-Glo™ and NADP/NADPH-Glo™ luminescence assays according to the manufacturer’s instructions (Promega). Total carbohydrates were extracted as described by Garcia et al. (2006). Soluble sugars were analyzed by HPAEC–PAD using a Dionex ICS-5000+ system (Thermo Fisher Scientific) following the method of Suksom et al. (2015), and starch content was determined from the insoluble fraction as described by Morris (1948) and Yemm and Willis (1954). Chlorophyll was extracted from frozen leaf tissue (100 mg) in 92% acetone, and absorbance was measured at 663 and 645 nm to calculate chlorophyll concentrations according to Arnon (1949). Total carotenoids were quantified at 470 nm using the same extracts.

### O_2_ consumption and ATP measurement

Oxygen consumption was measured using a high-resolution respirometry system (Oroboros O2k, Oroboros Instruments). Leaves from the same rosette position were collected from 35-day-old plants maintained in the dark for at least 1 h at room temperature. Four independent biological replicates were analyzed per genotype (n=4). Approximately 5 mg of fresh leaf tissue was excised, immersed in PBS, and transferred to the respirometry chamber. Measurements were performed in the dark to prevent photosynthetic O₂ evolution. Following signal stabilization, oxygen consumption was determined from the slope of a stable, approximately linear 3-min decrease in oxygen concentration. Rates were normalized to the actual fresh mass of each sample and expressed as [pmol/(s*mg FW)]. Leaf ATP content in 35-day-old plants was determined using an ATP assay kit (Invitrogen, Waltham, MA, USA) and a GloMax-Multi Detection System (Promega, Madison, WI, USA) essentially as described by Arcuri et al. (2021). Briefly, 50 mg of homogenized tissue powder was used for the extraction and assay.

### Statistical analysis

The data distribution and homogeneity of variances were assessed using Shapiro–Wilk and Levene's tests, respectively. For datasets that did not meet the normality assumption, Box–Cox transformation was applied to approximate a normal distribution. Once these assumptions were verified, statistical analyses were conducted using one-way or two-way ANOVA, depending on the experimental design, to assess the effects of genotype, treatment, and their interaction when applicable. When significant effects were observed, Dunnett's post hoc test was used to compare the mutant groups with the WT control group. The results are presented as the mean ± standard deviation (SD) or standard error of the mean (SEM), as indicated in the figure legends. A *p*-value of less than 0.05 was considered statistically significant. The graphs were designed using GraphPad Prism. The Holm-Sidak method for multiple comparisons against control was used to access interaction between genotype and light irradiance.

## Results

**Fig. 1:**
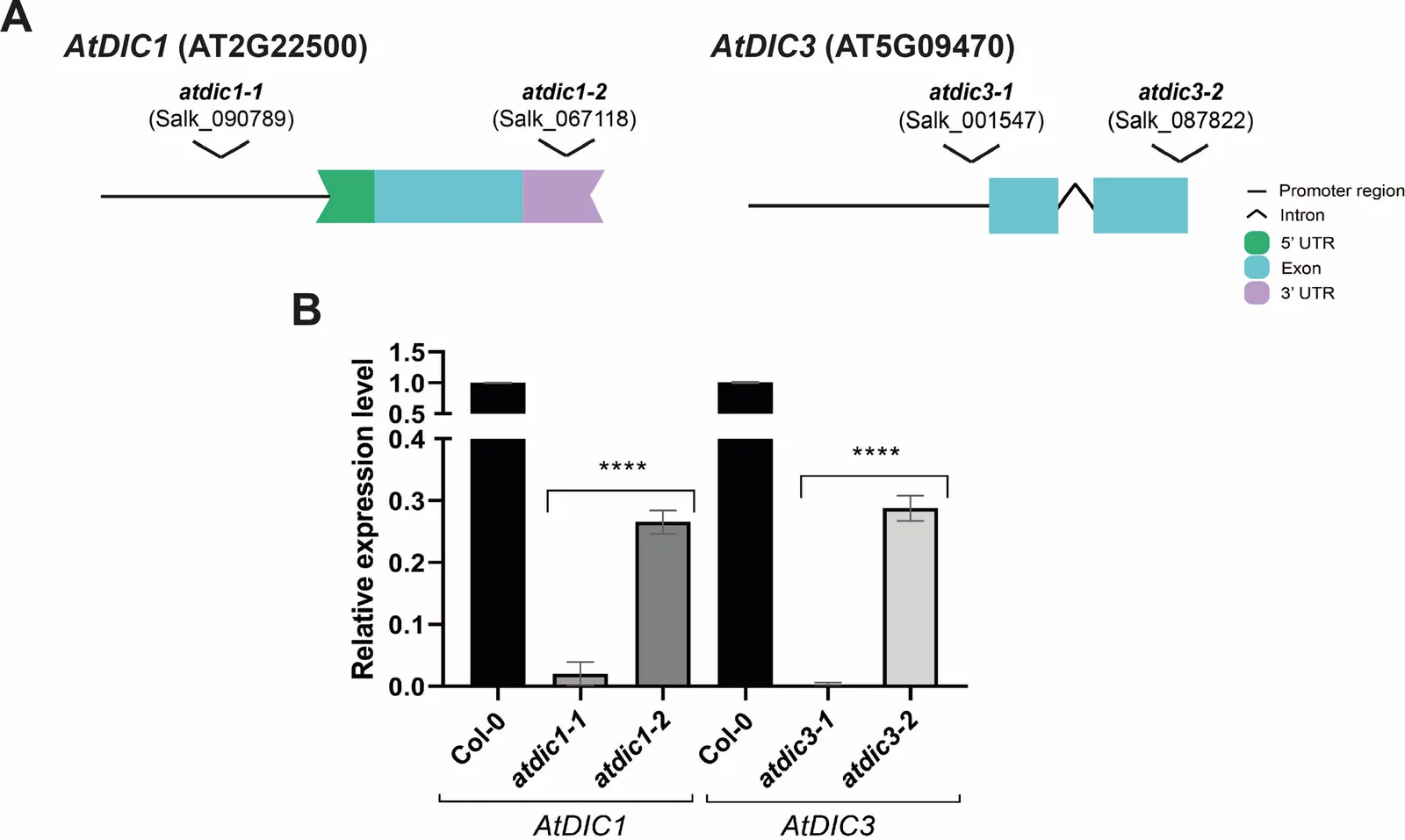
Molecular characterization of *atdic1* and *atdic3* T-DNA insertion mutants. (**A**) Gene structure of *AtDIC1* and *AtDIC3*, showing exons (boxes), introns (lines), and T-DNA insertion sites (arrows). (**B**) Relative expression of *AtDIC1* (using total RNA from seedlings) and *AtDIC3* (using total RNA from flowers) in homozygous mutant lines. Wild type Col-0 (WT) expression was used as a calibrator (set to 1). Asterisks indicate significant differences from WT expression as determined by one-way ANOVA followed by Tukey’s test (****p<0.0001)

To address the functional roles of *AtDIC1* and *AtDIC3* publicly available T-DNA insertion lines for each gene i.e. SALK_090789 and SALK_067118 for *AtDIC1*, and SALK_001547 and SALK_087822 for *AtDIC3*, were employed. Lines SALK_090789 (named *atdic1-1*) and SALK_067118 (*atdic1-2*) contained T-DNA insertions in the promoter [− 289 base pairs (bp) from the transcription start site (TSS)] and 3´untranslated region (+ 1542 bp from TSS) of *AtDIC1*, respectively (Fig. 1A). Lines SALK_001547 (*atdic3-1*) and SALK_087822 (*atdic3-2*) have annotated insertions in the proximal promoter (− 167 pb from TSS) and in the terminal portion of the coding region (+ 1390 pb from TSS) of *AtDIC3*, respectively (Fig. 1A). Insertion sites were confirmed by sequencing and homozygous individuals for each line were selected using PCR genotyping (Fig. S1).

Before checking the impact of the T-DNA insertions in target gene expression, we first examined the expression pattern of *AtDIC1*, *AtDIC2 a*nd *AtDIC3* in different organ/tissues of Arabidopsis using RT-qPCR. As shown in Fig. S2A, *AtDIC1* displayed a ubiquitous expression pattern with high transcripts levels being detected in rosette, floral buds at an early stage of development (stage 9; Boyes et al. 2001) and in flowers at the post-anthesis stage (stage 15; Boyes et al. 2001). *AtDIC2* was also ubiquitously expressed but at lower levels compared to *AtDIC1* and predominantly detected in stems, which corroborates previous transcriptome data (Klepikova et al. 2016). On the other hand, *AtDIC3* transcripts were detected at low levels and were relatively more abundant in post-anthesis flowers and in siliques, which suggest a more specialized function during the reproductive phase. Due to the proposed role of DIC in heterotrophic tissues (Palmieri et al. 2008), we also examined the expression of *AtDIC1-3* in germinating WT Col-0 seeds. To achieve this, seeds were collected at 1, 2, 3, and 4 DAS, concurrent with the light-mediated reactivation of seed metabolism (Rosental et al. 2014). As depicted in Fig. S2B, while *AtDIC1* and *AtDIC2* exhibited a steady increase in expression over the four time points analyzed, *AtDIC3* remained nearly unchanged until 4 DAS, when it reached its peak expression.

Transcript levels in each single mutant were then evaluated using total RNA extracted from the organ/tissues with the highest expression levels of the target genes *i.e. AtDIC1* in seedlings and *AtDIC3* in flowers. Compared to the WT (Col-0), the *atdic1-1* and *atdic3-1* lines showed a 97.9% and 99.9% reduction in the transcript levels of *AtDIC1* and *AtDIC3*, respectively, indicating an almost complete gene knockout (Fig. 1B). In contrast, *atdic1-2* and *atdic3-2* mutant lines exhibited a less pronounced reduction in the transcript levels of the genes targeted by T-DNA insertion, with decreases of approximately 73% for *AtDIC1* and 71% for *AtDIC3*, respectively (Fig. 1B). This likely reflects differences in the T-DNA insertion positions among these lines, which results in less severe transcriptional disruption.

### The germination of *atdic* mutant seeds is delayed at early stages

**Fig. 2:**
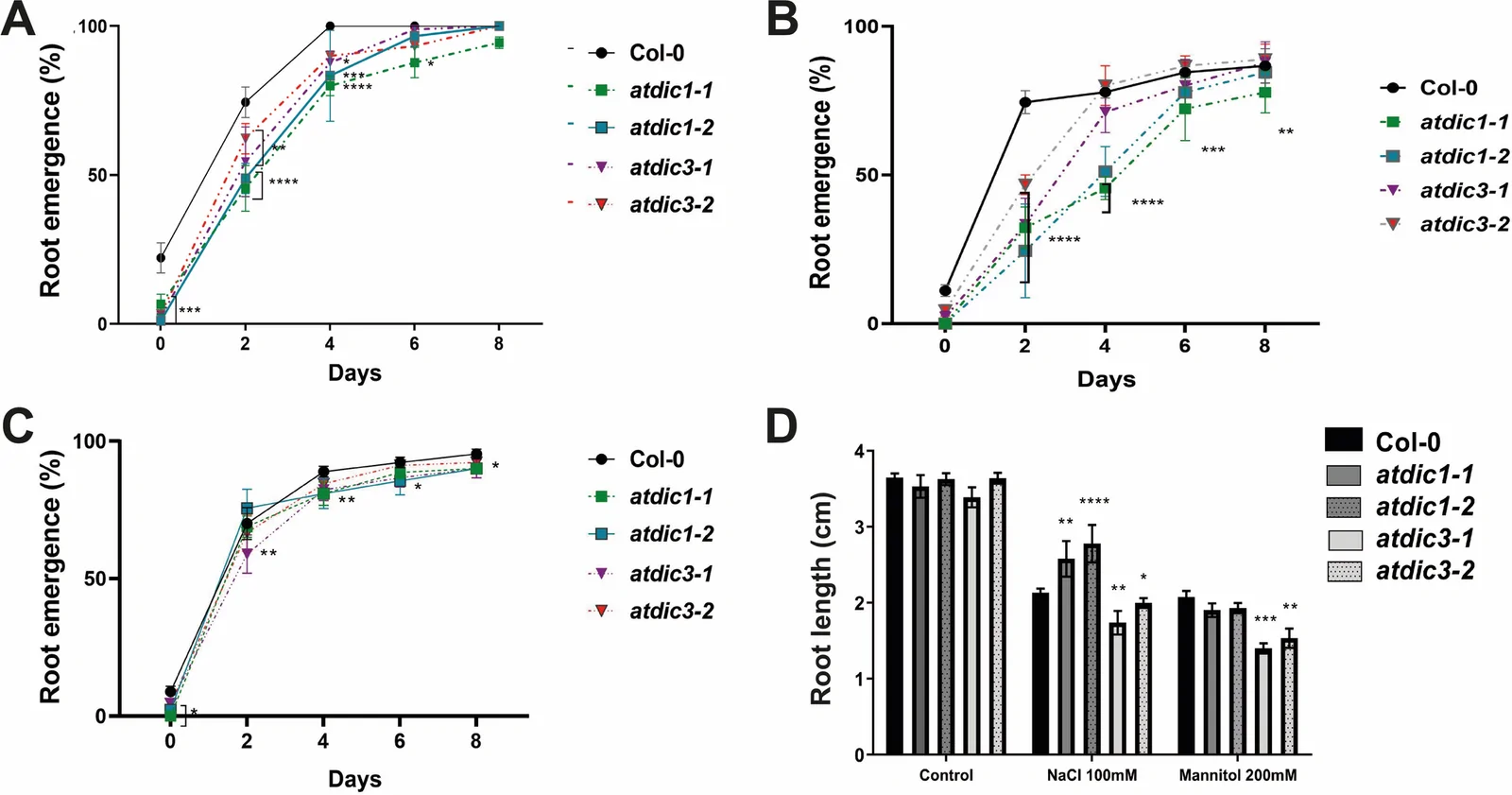
Impact of *AtDIC1* and *AtDIC3* downregulation on early Arabidopsis development. Germination rates (%) of wild type Col-0 (WT) and mutant seeds were assessed on 1/2 MS medium (with sucrose) under control condition (**A**) or supplemented with 200 mM mannitol (**B**) or 100 mM NaCl (**C**). Data represents the means of three independent assays (30 seeds per genotype per plate) measured over an 8-day period. (**D**) Primary root length of WT and mutant seedlings measured after 10 days of growth under control and stress conditions. Data are means ± SD of three independent assays (5 seedlings per genotype). Asterisks indicate significant differences from WT as determined by two-way ANOVA followed by Dunnett's multiple-comparisons test (*p<0.05, **p<0.01, ***p<0.001 and ****p<0.0001)

A potential role for DICs, particularly AtDIC1, in lipid reserve mobilization during seed germination has been proposed (Palmieri et al. 2008). To investigate whether the reduced expression of *AtDIC1* and *AtDIC3* would affect this process, we assessed the germination rate of WT and single mutant seeds (n=30/genotype) under control conditions (1/2 MS). Given the reported induction of *DIC* expression under various abiotic stresses (Barreto et al. 2022), we also evaluated seed germination in media supplemented with NaCl or mannitol to impose salt and osmotic stress, respectively. In the early stages following stratification (0–4 DAS), all mutant seeds exhibited delayed radicle emission under control conditions compared to WT, except for *atdic3-2* seeds at 4 DAS (Fig. 2A). These initial differences in germination rates diminished over time, with all mutant lines (except *atdic1-1*) approaching WT germination. Under osmotic stress, mutant seeds generally exhibited slower germination than WT at 2 DAS, except for *atdic3-2* (Fig. 2B). At subsequent time points, a significant delay in radicle emergence was observed only in *atdic1-1*, suggesting that impairment of *AtDIC1* had a more pronounced effect on germination kinetics under osmotic stress (Fig. 2B). Under salt stress, germination was delayed in the mutant lines relative to WT, although the magnitude and persistence of this delay varied among genotypes and over time (Fig. 2C). At 2 DAS, *atdic3-1* showed the most pronounced reduction in radicle emergence relative to WT, whereas the other mutant lines exhibited emergence rates closer to those of WT. At 4 DAS, all mutant lines showed lower or comparable radicle emergence relative to WT, with the strongest delay observed in the *atdic1* lines. By 6 DAS, the differences among genotypes were further reduced, with most mutant lines approaching WT germination. By 8 DAS, germination was high across all genotypes, and the differences were largely diminished, although a significant delay persisted in the *atdic1* lines (Fig. 2C). Overall, the differences among genotypes under salt stress were less pronounced than those observed under osmotic stress and were largely transient.

To assess the impact of carbon source availability on the germination of single mutant seeds, we evaluated their germination rate in 1/2 MS medium without sucrose. In the sucrose-free medium, the seeds of *atdic1-1* and *atdic1-2* exhibited both delayed germination and a lower final germination percentage compared to the WT (Fig. S3A), indicating limitations when no soluble sugar is supplied. When supplemented with mannitol or NaCl, delayed germination was observed exclusively in the *atdic1-1* mutant line, with no differences seen in the other mutants (Fig. S3B, S3C). Intriguingly, *atdic3-2* seeds displayed a mild improvement in germination rate under osmotic stress at 1 DAS relative to the WT (Fig. S3B). Overall, these results support the hypothesis that these carriers, particularly AtDIC1, play a relevant role in the early stages of seed germination, especially under stress conditions.

### Single mutants exhibit changes in root growth under stress

The potential impacts of the reduced expression of the target genes on initial seedling growth were assessed by determining primary root growth under normal and stressful conditions. Interestingly, despite the delayed seed germination rate noticed in the mutant lines (Fig. 2), the roots of all tested lines developed normally under control growth conditions and no significant effect on primary root length compared to the WT was observed (Fig. 2D). Under osmotic or salt stress conditions, both the *atdic3-1* and *atdic3-2* lines exhibited a significant reduction in primary root length, indicating that AtDIC3, despite its unique expression pattern, may play a relevant role in the stress response during this developmental stage (Fig. 2D). Meanwhile, primary roots of *atdic1* mutants were significantly longer than those of the WT under salt stress, whereas no significant differences were observed under mannitol treatment, despite a trend toward decreased root length in the mutants (Fig. 2D).

### Single mutant plants display increased biomass accumulation

**Fig. 3:**
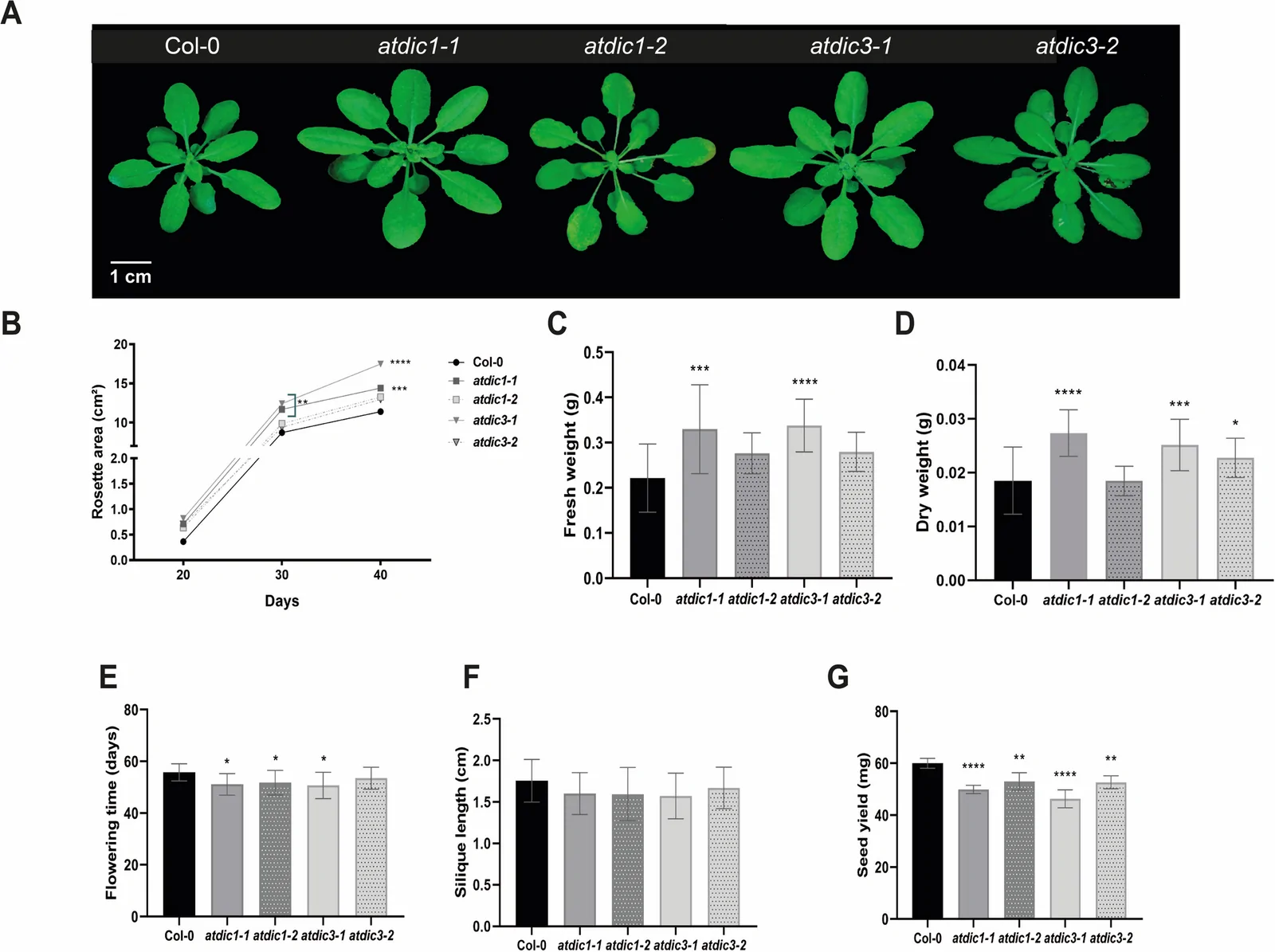
Phenotypic analysis of *atdic1* and *atdic3* plants. (**A**) Representative images of long-day grown plants (40 DAS) from wild type Col-0 (WT) and mutant lines. (**B**-**D**) Rosette-related parameters: rosette area over time (**B**) and mean rosette fresh (**C**) and dry (**D**) weights per plant at 40 DAS; (**E**–**G**) Reproductive parameters: flowering time (**E**), silique length (**F**), and seed yield per plant (**G**). Asterisks indicate significant differences from WT as determined by one-way ANOVA followed by Tukey’s test (*p<0.05, **p<0.01, ***p<0.001 and ****p<0.0001)

The vegetative development of the *atdic1* and *atdic3* mutant lines was assessed by measuring the rosette area over time and the aboveground biomass (fresh and dry). Surprisingly, the mutant plants exhibited increased growth compared to WT plants (Fig. 3A). This improved growth was accompanied by significant increases in rosette area over time, which became noticeable by 30 DAS and was particularly pronounced at 40 DAS (Fig. 3B). At this time point, plants from the *atdic1-1* and *atdic3-1* lines, which exhibited the greatest reduction in target gene expression, accumulated significantly more biomass, both fresh and dry weight, than the WT (Fig. 3C and D). Although not statistically significant, a similar trend of increased fresh weight was observed in the *atdic1-2* and *atdic3-2* lines, likely due to the lower levels of gene knockdown in these lines. Subsequently, the differences observed gradually diminished and were no longer perceptible. Of note, the same whole-plant phenotypes were observed in short-day growth conditions (not shown). During the reproductive phase, all mutant lines, except *atdic3-2*, exhibited an early-flowering phenotype, flowering 3 and 4 days earlier, respectively, compared to WT plants (Fig. 3E). Additionally, while producing normal siliques (Fig. 3F), a significant reduction in total seed yield was observed in all mutant lines compared to the WT (Fig. 3G). These results suggest a metabolic role for these carriers during seed production.

To further investigate the functional consequences of reduced *DIC* expression, we concentrated our efforts on the lines exhibiting the highest levels of gene knockdown, specifically *atdic1-1* and *atdic3-1*, which are likely null alleles. These lines will hereafter be referred to as *atdic1* and *atdic3*, respectively.

### Net photosynthesis is increased in the *atdic* single mutants

**Fig. 4:**
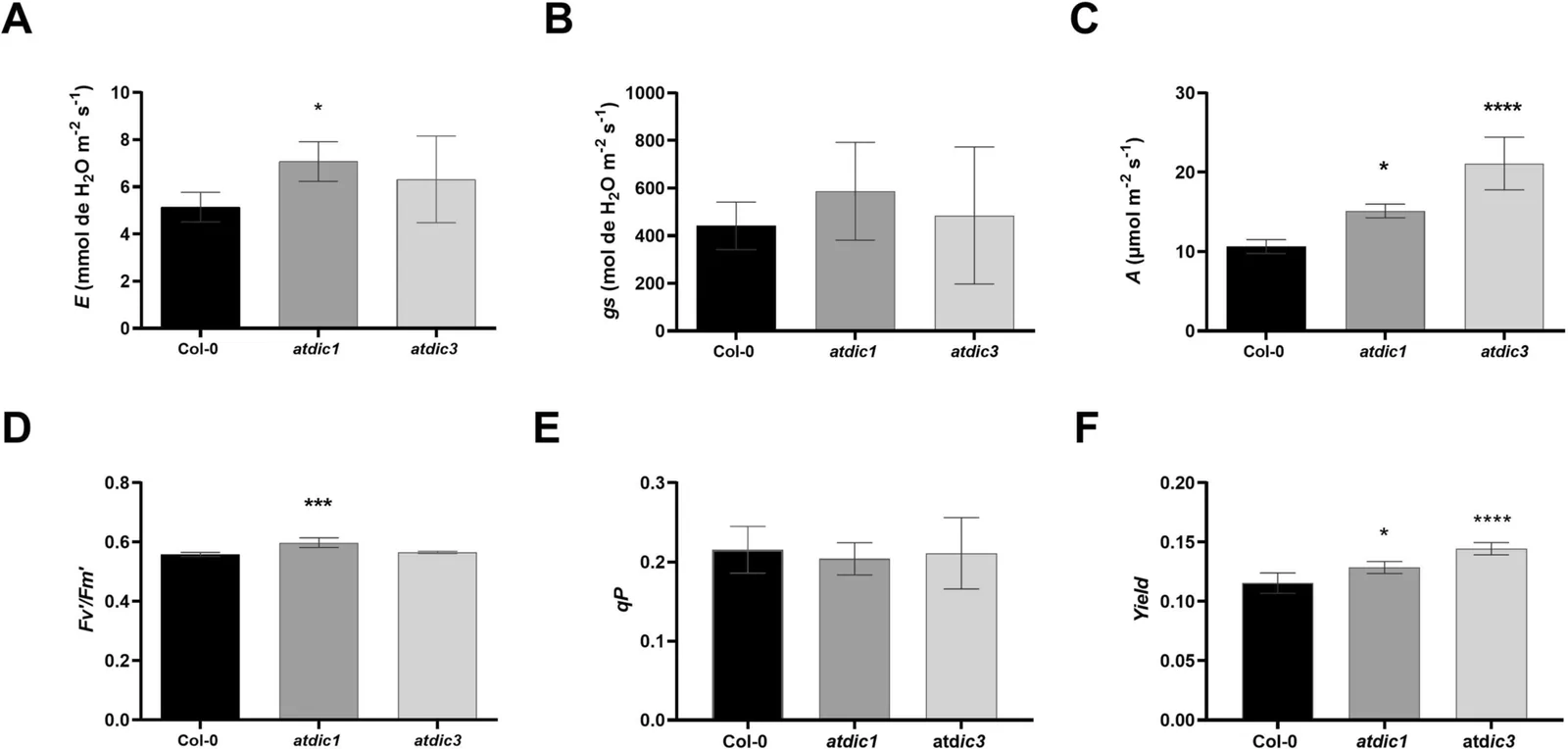
Photosynthetic parameters and chlorophyll fluorescence in *atdic1* and *atdic3* mutant plants. (**A**) transpiration rate (*E*); (**B**) stomatal conductance (*gs*); (**C**) net CO₂ assimilation rate (*A*); (**D**) PSII maximum efficiency in the light-adapted state (*Fv’/Fm’*); (**E**) photochemical quenching (*qP*) and (**F**) effective quantum efficiency of PSII (*Y*). Asterisks indicate significant differences from wild type Col-0 as determined by one-way ANOVA followed by Tukey’s test (*p<0.05; ***p<0.001 and ****p<0.0001)

Given that photosynthesis accounts for the majority of plant biomass, we investigated whether gene knockout influenced the photosynthetic efficiency of the single mutant plants (35 DAS). As shown in Fig. 4, significant differences in gas exchange and chlorophyll fluorescence parameters were observed between the WT and the single mutants. Mutants exhibited a trend toward increased transpiration rates (*E*) compared with the WT; however, this increase was statistically significant only in *atdic1* (Fig. 4A). In contrast, stomatal conductance (*gs*) did not differ among genotypes (Fig. 4B). Consistent with improved photosynthetic capacity, both mutants showed significantly higher net CO₂ assimilation rates (*A*_*n*_), with the strongest increase observed in *atdic3* (Fig. 4C). Chlorophyll fluorescence analysis showed that only *atdic1* exhibited an increase in *Fv′/Fm′* compared with the WT, whereas no differences were observed in *qP* among genotypes (Fig. 4D, E). Notably, both mutants displayed significantly higher Y(II) (or Φ_PSII_) relative to the WT (Fig. 4F), which was accompanied by increased electron transport rates (*ETR*; data not shown). These results indicate that the enhanced photosynthetic performance of *atdic1* and *atdic3* is primarily associated with improved photochemical efficiency and increased electron transport activity, rather than changes in stomatal conductance. The increased biomass observed in these mutants (Fig. 3) coincides with higher photosynthetic capacity and increased *A*_*n*_, particularly in *atdic3*, suggesting a link between photosynthetic performance and biomass accumulation.

### *AtDIC2* expression is highly induced in the *atdic1* and *atdic3* mutants

**Fig. 5:**
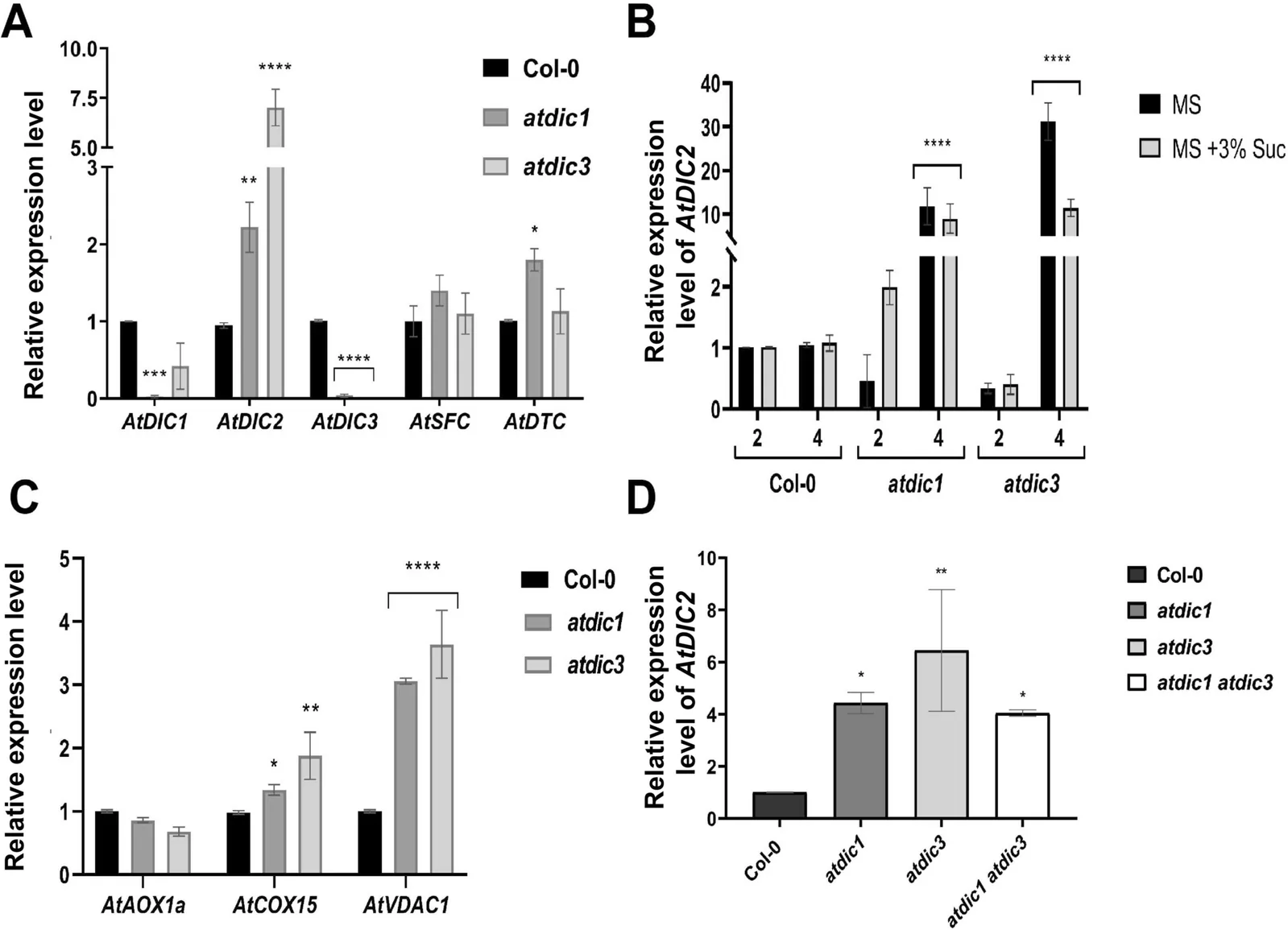
Relative gene expression analysis. (**A**) *AtDIC1, AtDIC2, AtDIC3*, *AtSFC* and *AtDTC* expression in wild type Col-0 (WT) and single mutant plants; (**B**) *AtDIC2* expression in WT and single mutant seeds at 2 and 4 days after stratification (DAS); (**C**) Expression of mitochondrial dysfunction marker genes (*AtAOX1a*, *AtCOX15* and *AtVDAC1*) in WT and single mutant plants; (**D**) *AtDIC2* expression in WT, single and double mutant plants. WT expression was used as a calibrator (set to 1). Asterisks indicate significant differences from WT as determined by one-way ANOVA followed by Tukey’s test (*** p<0.001 and ****p<0.0001)

To assess whether the reduced expression of the target genes in the mutant lines could be compensated at the transcriptional level by the other isoforms, we initially measured the transcript levels of the three *AtDIC* genes in 35-day-old plants of each mutant background. In parallel, we evaluated the relative expression of other members of the Mitochondrial Carrier Family (MCF) capable of transporting dicarboxylates, namely the succinate/fumarate transporter (SFC; At5g01340) and the di-tricarboxylate transporter (DTC; At5g19760) (Nunes-Nesi et al. 2020). In both *atdic1* and *atdic3* mutants, *AtDIC2* transcript levels were significantly increased compared to the WT, consistent with a potential compensatory response (Fig. 5A). This induction was particularly pronounced in the *atdic3* mutant (approximately 6–7-fold relative to Col-0). Interestingly, the transcriptional response was divergent: while *AtDIC2* was induced, the transcript levels of the other isoforms (*AtDIC3* in *atdic1*, and *AtDIC1* in *atdic3*) remained low (Fig. 5A). These results suggest that, under normal growth conditions, the increased expression of *AtDIC2* may act as a primary compensatory mechanism in these single mutants. Complementary analysis showed that the transcript levels of *AtSCF* remained unchanged in mutants compared to the WT, whereas a sharp increase in *AtDTC* transcript levels was observed exclusively in *atdic1* (Fig. 5A). Because their expression was not consistently affected across both single mutants, unlike the robust response of *AtDIC2*, these other MCF members are unlikely to play a central role in a generalized compensatory mechanism.

Given the observed delay in germination phenotype (Fig. 2), we also investigated whether *AtDIC2* expression is altered during seed germination. Consistent with the results observed during the vegetative stage, *AtDIC2* transcript levels were significantly higher at 4 DAS in both mutant seeds compared to WT, regardless of whether seeds were sown on 1/2 MS medium with or without sucrose (Fig. 5B). In addition, a non-significant trend toward increased *AtDIC2* transcript accumulation was observed at 2 DAS in *atdic1* seeds germinated on 1/2 MS medium supplemented with sucrose. However, unlike the hypothesized compensatory complementation during vegetative development, the observed upregulation appears insufficient to offset the germination defects associated with the reduced expression of the target genes, particularly *AtDIC1*.

### Impacts on mitochondrial function

To investigate the impact of reduced *AtDIC1* or *AtDIC3* expression on mitochondrial function, we examined the expression levels of marker genes indicative of the mitochondrial status, including *AOX1a* (Alternative oxidase 1a; At3g22370), *COX15* (Cytochrome C oxidase 15; At5g56090), and *VDAC1* (Voltage-dependent anion channel 1; At3g01280; Pan et al. 2014), in the tested single mutant lines. Both *atdic1* and *atdic3* mutants showed increased transcript levels of *VDAC1* and *COX15* (Fig. 5C). It is worth mentioning that *COX15* is upregulated (3.2-fold) in response to rotenone treatment, a well-established inhibitor of the electron transport chain (Lister et al. 2004). In contrast, *AOX1a* expression in the mutants was not significantly altered compared to WT (Fig. 5C), indicating that the alternative respiratory pathway was not induced at the transcriptional level. Although not exhaustive, these results suggest that while the expression of certain components involved in cellular energy regulation and mitochondrial function, such as VDAC1 and COX15, is affected by the reduced expression of *AtDIC1* and *AtDIC3*, AOX1a appears to be only minimally impacted under these conditions. However, we cannot exclude the possibility of AOX1a activation via post-translational modifications or protein stabilization, which are common regulatory mechanisms for the alternative respiratory pathway (Maxwell et al. 1999; Vanlerberghe et al. 2013).

### Metabolic changes associated with the reduced expression of *AtDIC1* and *AtDIC3*

**Fig. 6:**
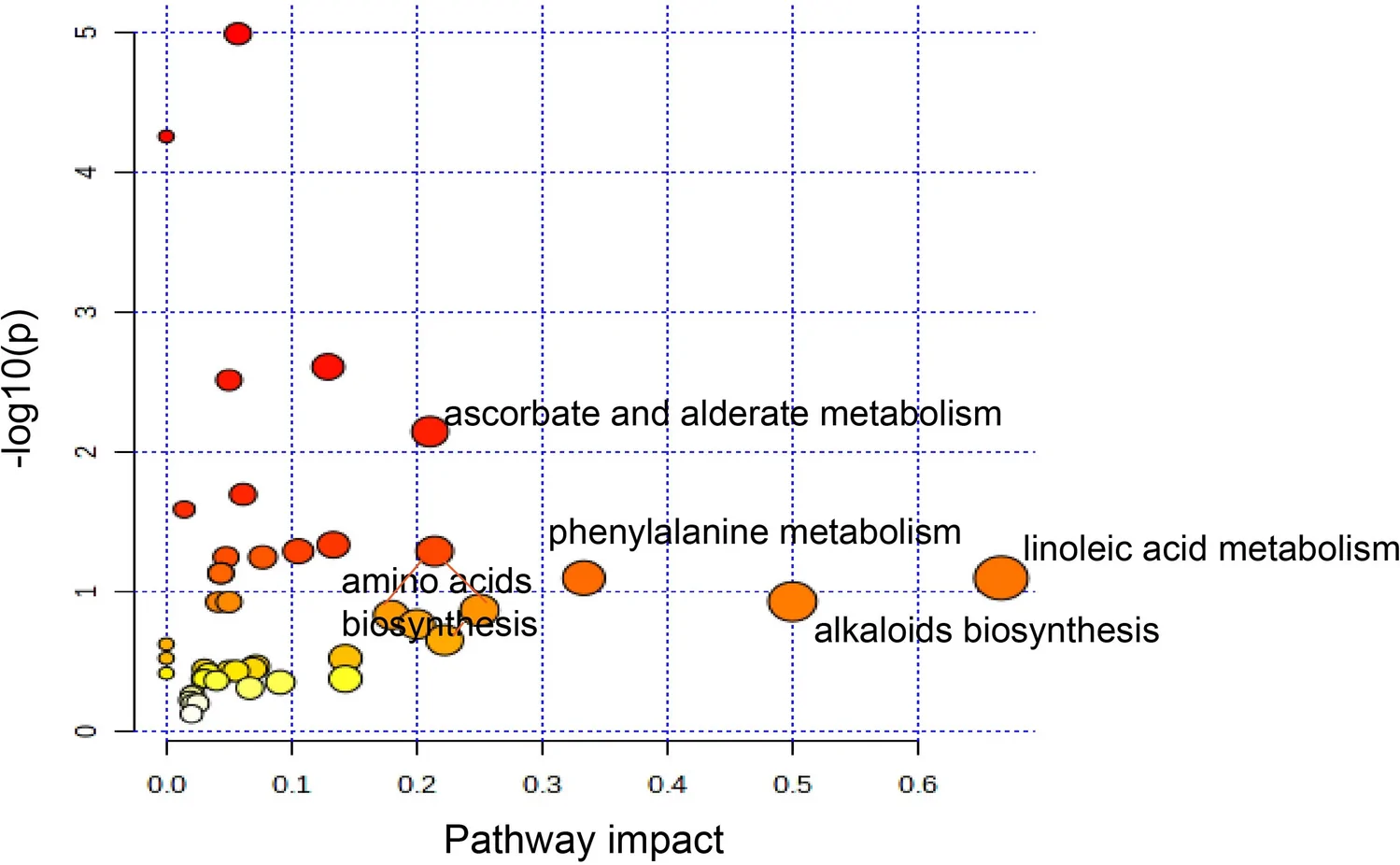
Altered metabolic pathways between WT Col-0 and single mutants. The metabolic pathways are depicted as circles according to their scores from enrichment (y-axis) and topological (x-axis) analyses. The size of the circle corresponds to the pathway impact score and is associated with the centrality of the implicated metabolites; the color displays the significance (darker circles indicate more significant changes). Only the metabolic pathways with p < 0.05 are depicted

To identify metabolic changes associated with reduced expression of *AtDIC1* and *AtDIC3*, a non-targeted metabolomic analysis was performed. Considering the unexpected increase in biomass observed in the mutant plants, rosettes from 35 DAS plants were selected for analysis. After data normalization, a total of 102 metabolites were detected across the sample set. Carbohydrates and their derivatives represented the largest class (37.6%), followed by lipids (13.4%), organic acids (11.8%), organometallic compounds (11.0%), and amino acids (10.5%). Of these, 86 metabolites were differentially accumulated in the single mutants compared with the WT (Table S2). Figure S4 presents a heatmap of the 50 most significantly altered rosette metabolites, with the most abundant compounds highlighted in red. Hierarchical clustering revealed genotype-specific metabolic profiles: *atdic1* displayed higher sugar accumulation, whereas *atdic3* was characterized by increased amino acid levels. Noteworthy, *atdic3* displayed approximately 3-fold higher levels of serine, alanine, and proline compared with *atdic1*. Pathway enrichment analysis identified linoleic acid metabolism, alkaloid biosynthesis, phenylalanine metabolism, ascorbate and aldarate metabolism, and amino acid biosynthesis as the most significantly affected pathways, with impact scores of 0.66, 0.50, 0.25, 0.23, and 0.22, respectively (Fig. 6). Disruption of key metabolites within the phenylalanine metabolism pathway was observed in both *atdic1* and *atdic3*, whereas alterations in ascorbate and aldarate metabolism were primarily associated with *atdic1*. In this context, ascorbate levels increased by 2.33-fold in *atdic1* and 1.90-fold in *atdic3* relative to the WT (Table S2). In contrast, changes in arginine and proline metabolism were specific to *atdic3*.

### Analysis of the double mutant *atdic1/atdic3*

**Fig. 7:**
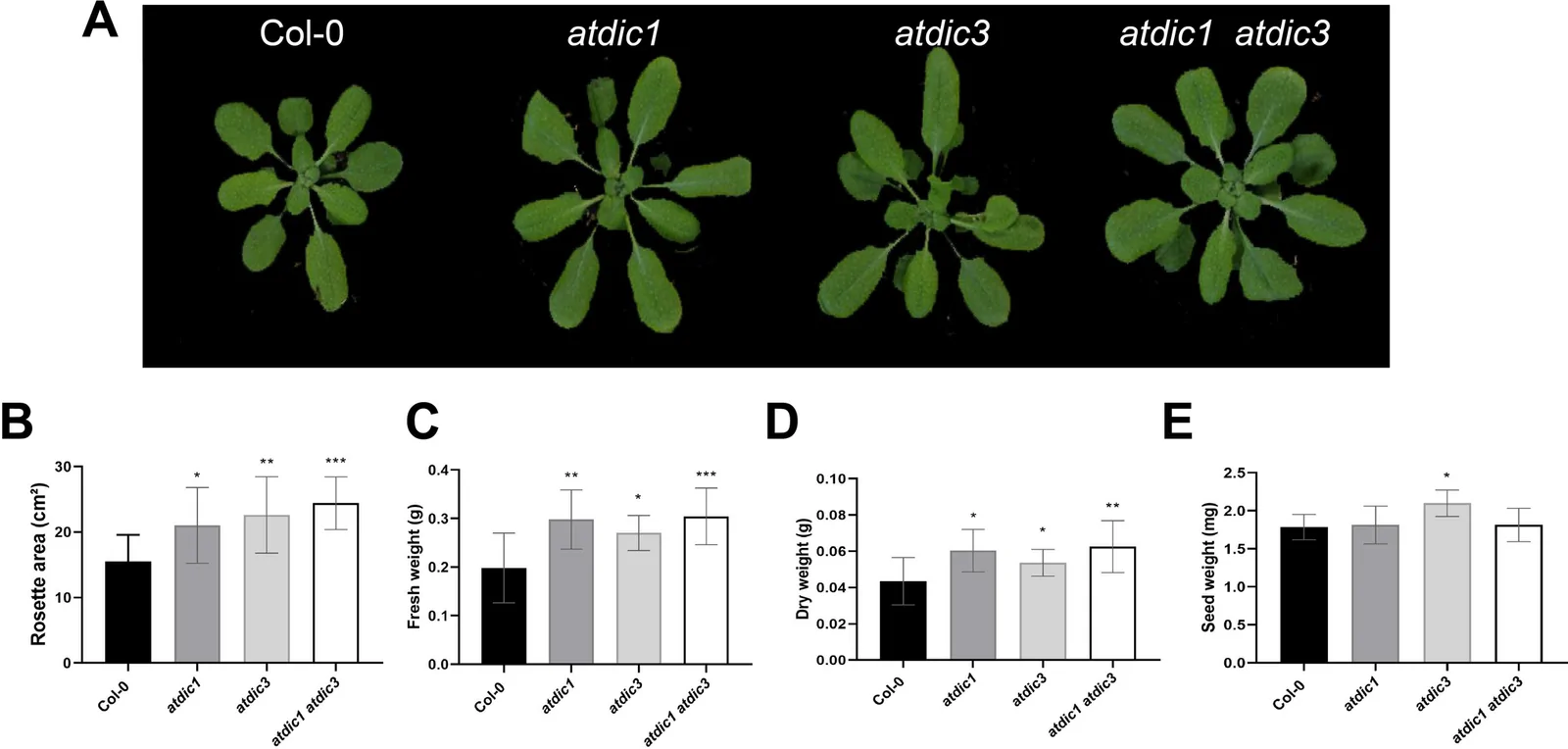
Phenotypic analysis of *atdic1/atdic3* double mutant plants. (**A**) Representative images of 40-day-old wild type Col-0 (WT), *atdic1*, *atdic3*, and *atdic1/atdic3* plants; (**B**) Rosette area; mean rosette fresh (**C**) and dry (**D**) weights per plant at 40 DAS and (**E**) 100-seed weight. Asterisks indicate significant differences from WT as determined by one-way ANOVA followed by Tukey’s test (*p-<0.05, **p<0.01, and ***p<0.001)

To investigate the effect of combined mutations at *AtDIC1* and *AtDIC3*, a double mutant (termed *atdic1/atdic3*) was generated. The growth parameters of double mutant plants were evaluated at 40 DAS, the developmental stage at which the single mutant phenotypes were more pronounced (Fig. 7). Consistent with the single mutant phenotypes (Fig. 3), the double mutant exhibited a larger rosette area relative to the WT (Fig. 7A, B). The observed difference in rosette area was further supported by significantly higher fresh and dry biomass in the double mutant compared to the WT (Fig. 7C, D). In contrast, no significant differences in 100-seed weight were detected between the mutants and the WT, except for *atdic3*, which showed a significantly higher seed weight (Fig. 7E). Overall, the growth phenotype of the double mutant closely resembled that of the single mutants, with no statistically significant differences observed for the analyzed parameters, except for 100-seed weight, in which *atdic3* differed from the others (Table S3). These findings indicate that under the tested growth conditions, the simultaneous mutation of both genes did not result in a clear additive effect. Noteworthy, the double mutant also exhibited a significant increase in *AtDIC2* transcript levels relative to the WT, mirroring the pattern observed in the single mutants (Fig. 5D).

**Fig. 8:**
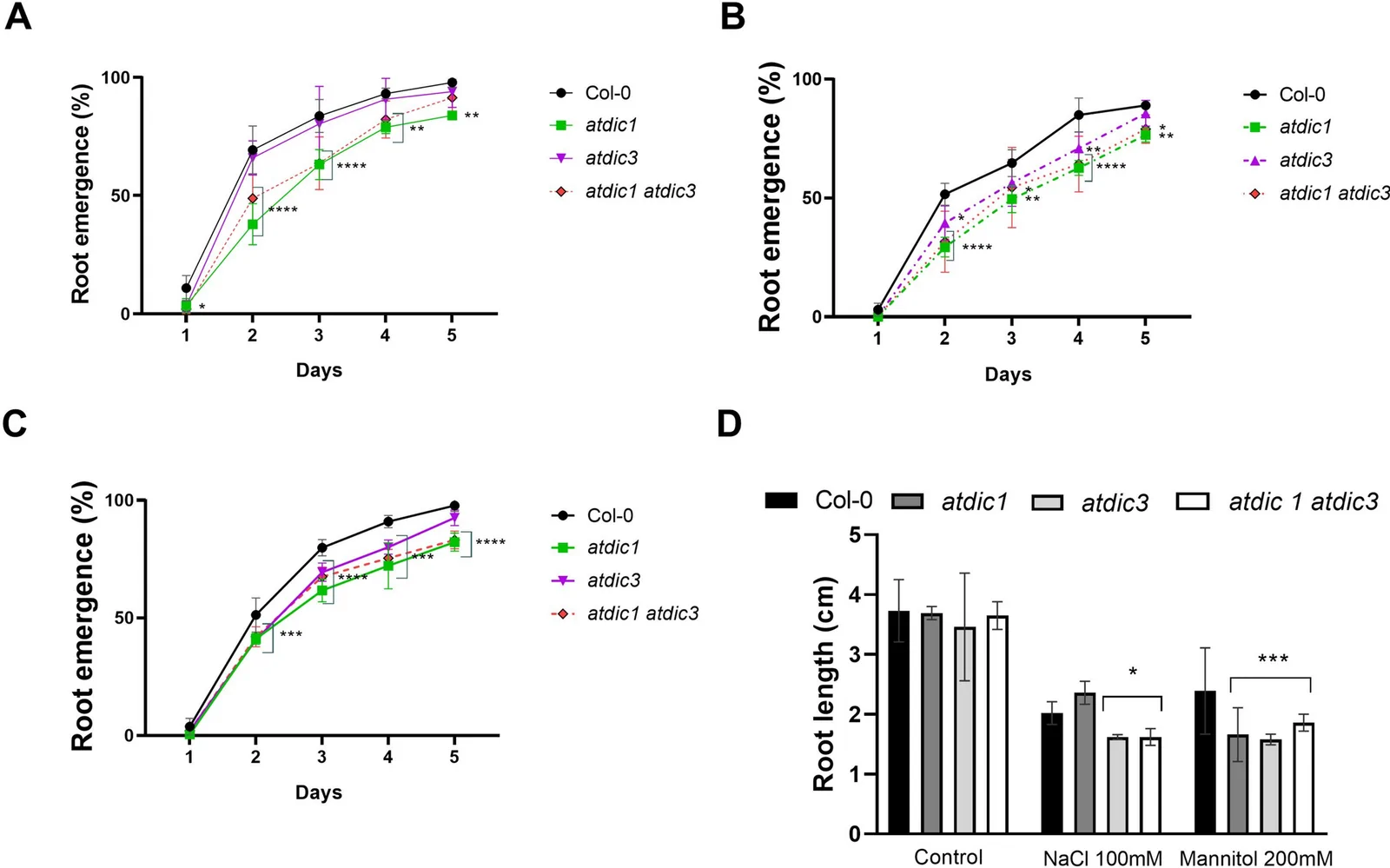
Effects of *AtDIC1* and *AtDIC3* double mutation on early Arabidopsis development. Germination rates (%) of mutant and wild type Col-0 (WT) seeds sown on 1/2 MS medium under control condition (**A**) or supplemented with 200 mM mannitol (**B**) or with 100 mM NaCl (**C**). Data represents the means of three independent assays (30 seeds per genotype per plate) measured over a 5-day period. (**D**) Primary root length of WT and mutant seedlings measured after 10 days of growth under control and stress conditions. Data are means ± SD of three independent assays (5 seedlings per genotype). Asterisks indicate significant from WT as determined by two-way ANOVA followed by Dunnett's multiple-comparisons test (*p<0.05, **p<0.01, ***p<0.001 and ****p<0.0001

To determine whether the *AtDIC1* and *AtDIC3* double mutation also affects early development, we assessed the germination rate (scored as radicle emergence) and primary root growth of the single and double mutants. Under control conditions, *atdic1* and *atdic1/atdic3* mutant seeds exhibited delayed radicle emission from 2 to 4 DAS, whereas *atdic3* remained comparable to the WT (Fig. 8A). By 5 DAS, however, only *atdic1* displayed a statistically significant delay compared to WT (Fig. 8A). Mutant seed germination was also consistently delayed under both salt and osmotic stress conditions. Under osmotic stress, all mutants exhibited a significantly lower germination percentage compared to WT across all examined time points, except for *atdic3* at 5 DAS (Fig. 8B). A similar pattern was also observed under salt stress (Fig. 8C). Taken together, and consistent with the results obtained for the single mutants (Fig. 2), these findings indicate that *atdic1* seeds exhibit the most pronounced delay in germination, with the *atdic1/atdic3* double mutant showing a broadly similar phenotype, whereas *atdic3* displays a milder, intermediate response. In agreement with our previous observations (Fig. 2D), primary root length under control conditions did not differ significantly between WT and any of the single or double mutants (Fig. 8D; Fig. S5A). In contrast, salt stress significantly reduced root length in the *atdic3* and *atdic1/atdic3* mutants compared with WT plants, whereas *atdic1* showed no significant difference from WT (Fig. 8D; Fig. S5B). Under osmotic stress, all mutants displayed a significant decrease in primary root length compared with WT (Fig. 8D; Fig.S5C).

### Single and double mutants exhibit enhanced photosynthetic pigment accumulation and increased PSII efficiency

To explore the factors contributing to the increased biomass accumulation observed in the mutants, we quantified photosynthetic pigment contents in leaves of the single and double mutants, given the central role of these pigments in light harvesting. On a fresh weight basis, total chlorophyll and chlorophyll *a* contents were significantly higher in all mutants compared with the WT (Fig. S6A, B). Distinct patterns were observed for the other pigments: chlorophyll *b* levels increased specifically in *atdic1* and the *atdic1/atdic3* double mutant (Fig. S6C), resulting in a higher chlorophyll *a/b* ratio in these lines (Fig. S6D). Conversely, carotenoid levels were significantly increased in *atdic3* and *atdic1/atdic3* (Fig. S6E).

To further assess the photosynthetic light reactions, we analyzed light-response curves for Φ_PSII_, *qP*, and *ETR* in the single and double mutants (Table S4). Compared to WT, all *atdic* mutants exhibited significantly higher *Φ*_*PSII*_ and *ETR*, indicating enhanced photochemical performance. The *atdic1* mutant also showed increased *qP*, whereas the double mutant displayed reduced *NPQ*. In addition, *atdic3* and *atdic1/atdic3* exhibited higher *Fv′/Fm′* values and lower heat dissipation (*D*), consistent with improved PSII efficiency under light-adapted conditions. No significant differences among genotypes were observed for excitation pressure (*Ex*). All genotypes followed a characteristic light-response pattern, where increasing PPFD significantly affected all fluorescence parameters: Φ_PSII_ and *qP* decreased, whereas *ETR* increased steadily across the entire light range. At higher PPFDs, *NPQ* (from 625 µmol photons m⁻^2^∙s⁻^1^ onward) and *D* (from 845 µmol photons m⁻^2^∙s⁻^1^ onward) increased, while *Fv′/Fm′* (from 845 µmol photons m⁻^2^∙s⁻^1^ onward) decreased, reflecting enhanced photoprotective energy dissipation under excess light. In contrast, no significant Genotype × PPFD interaction was observed for any of the fluorescence parameters, indicating that genotypes differed mainly in baseline photosynthetic performance rather than in their response to increasing light intensity.

### Redox status and carbohydrate metabolism are perturbed in mutants

To determine if cellular redox balance is affected by single and double mutations in *AtDIC1* and *AtDIC3*, we quantified dinucleotide pools and their respective redox ratios. A significant reduction in the NADH/NAD⁺ ratio, indicative of a more oxidized mitochondrial redox state, was observed in *atdic3* and *atdic1/atdic3* mutants compared to the WT, whereas no change was detected in *atdic1* (Fig. S7A). Conversely, the NADPH/NADP⁺ ratio was significantly increased in both *atdic3* and *atdic1/atdic3* mutants compared with the WT, while *atdic1* exhibited a non-significant increase (Fig. S7B). The elevated NADPH/NADP⁺ ratio is consistent with enhanced photosynthetic electron supply and increased reductive capacity to support biosynthetic processes and antioxidant systems. Collectively, these findings indicate that cellular redox homeostasis is specifically altered upon loss of AtDIC3, supporting a prominent role for this isoform in maintaining redox balance and suggesting that it is epistatic to AtDIC1 in this context.

Given these redox alterations, we subsequently analyzed primary carbon metabolism by measuring representative carbohydrate levels in leaves harvested mid-light period (Fig. S7). Sucrose levels were markedly reduced in all single and double mutants compared to the WT (Fig. S7C). In contrast, all mutant lines consistently accumulated significantly higher starch levels than the WT (Fig. S7D). These results demonstrate that the loss of DIC function leads to clear disturbances in carbon partitioning.

### Impacts on O_2_ consumption and ATP content

To assess potential alterations in respiratory metabolism, O_2_ consumption by leaf samples from WT and mutant plants was monitored in the dark using high-resolution respirometry. Oxygen flux showed a progressive increase in the mutant lines compared to WT, reaching statistical significance in *atdic3* and *atdic1/atdic3* (Fig. S8A). Consistent with the enhanced respiratory flux, cellular ATP content was significantly higher in *atdic3* leaves than in WT (Figure S8B). Although the *atdic1/atdic3* double mutant also showed an increase in ATP levels, this difference did not reach statistical significance. In contrast, ATP content in *atdic1* remained comparable to that of WT (Figure S8B). Thus, *atdic3* uniquely combined enhanced respiratory activity with increased ATP accumulation, suggesting a distinct adjustment of mitochondrial energy metabolism.

## Discussion

Dicarboxylate transport plays a critical role in multiple aspects of plant metabolism, growth, and stress responses. Among the Arabidopsis DIC family members, AtDIC2 has been functionally characterized and shown to be essential for mitochondrial malate–citrate exchange in vivo (Lee et al. 2021). In contrast, direct functional evidence for the other isoforms has remained limited. Among these, AtDIC1 is the most abundant, with approximately 59 protein copies per mitochondrion compared to 29 copies of AtDIC2 (Fuchs et al. 2020). AtDIC3, by contrast, is less abundant and exhibits a more restricted expression pattern, suggesting a more specialized physiological role. Supporting functional divergence among DIC isoforms, a recent study demonstrated that neither *AtDIC1* nor *AtDIC3* can compensate for the loss of *AtDIC2* in a *dic2* knockout background (Lee et al. 2021).

Early studies proposed that DICs may contribute to storage lipid mobilization during seed germination and/or gluconeogenesis (Palmieri et al. 2008). Here, we provide evidence that reduced expression of *AtDIC1* and *AtDIC3* impairs seed germination under both control and stress conditions. This phenotype, however, was most pronounced in *atdic1* seeds, particularly when germinated on sucrose-free medium, indicating a compromised capacity to mobilize endogenous carbon reserves. These results identify AtDIC1 as a key component in maintaining metabolic flexibility during germination, a stage characterized by rapidly shifting energy and biosynthetic demands (Rosental et al. 2014). During this transition, mitochondria are rapidly reactivated prior to the establishment of photosynthetic competence, requiring tight coordination between reserve mobilization, respiratory metabolism, and redox balance. In this context, impaired dicarboxylate transport may limit metabolic flexibility during germination, particularly under stress conditions that impose additional energetic demands. Consistent with this, our expression analyses revealed a high *AtDIC1* transcript accumulation during early germination (1–4 DAS), coinciding with the reactivation of seed metabolism (Rosental et al. 2014), whereas *AtDIC3* expression increased only at later stages. The absence of a stronger germination defect in the *atdic1/atdic3* double mutant further supports a dominant contribution of AtDIC1 during this developmental window. In this regard, the inability of *AtDIC2* upregulation to fully rescue the observed germination defects suggests that, despite transcriptional compensation, AtDIC2 is unable to fully replace AtDIC1 during early seed development. Consistent with the broader role of mitochondrial function in developmental regulation, several studies have shown that mitochondrial dysfunction can influence hormone homeostasis and signaling (Welchen et al. 2021). For example, *Cytochrome c* mutants exhibit delayed seed germination and, in at least one reported case, altered abscisic acid sensitivity (Meyer et al. 2009; Racca et al. 2022; Chen et al. 2024). Conversely, overexpression of the transcription factor ERF1, a central component of the canonical ethylene signaling pathway, was shown to promote seed germination by enhancing mitochondrial function (Chen et al. 2026). Together, these findings highlight a close interplay between mitochondrial activity and hormonal regulation during germination. Investigating whether similar regulatory crosstalk occurs in *atdic* mutants could therefore provide important insights into how mitochondrial transporters integrate metabolic and hormonal cues during early seed development.

Primary root growth analyses revealed a divergence between *AtDIC1* and *AtDIC3* under stress conditions. The *atdic3* mutant consistently exhibited reduced root elongation under both salt and osmotic stress, indicating a clear stress-sensitive phenotype. In contrast, *atdic1* showed normal or even enhanced root growth under salt stress, whereas under osmotic stress, a significant reduction in root growth was observed in only one of the two independent experiments. Thus, the variable response of *atdic1* across stress conditions and independent experiments precluded a clear conclusion regarding its stress sensitivity. A contrasting stress-dependent response was also observed in the mitochondrial *ndufs4* mutant, which lacks the 18-kDa subunit of Complex I (Meyer et al. 2009). Despite exhibiting an overall delayed growth phenotype, this mutant showed enhanced root growth tolerance relative to the WT plants under cold, osmotic, and moderate salt stress conditions (Meyer et al. 2009). Collectively, our findings suggest that AtDIC3 plays a predominant role in sustaining root growth under adverse conditions, potentially by preserving mitochondrial metabolic flexibility and redox homeostasis in actively dividing root cells. Although *AtDIC3* is predominantly expressed in reproductive tissues, it is also highly responsive to abiotic stress and has been identified as the most consistently induced DIC isoform under stress conditions (Kilian et al. 2007). In addition, *AtDIC3* expression is tightly regulated by inorganic phosphate availability, being strongly induced under phosphate starvation and repressed in mutants defective in phosphate homeostasis regulators (Bari et al. 2006; Barreto et al. 2022). Thus, despite its relatively low basal expression, AtDIC3 may be selectively recruited under stress conditions to enhance metabolic flexibility. As a putative malate–citrate antiporter, AtDIC3 may facilitate cytosolic malate import into mitochondria, thereby supporting inter-organellar malate circulation and stress mitigation, as proposed previously (Zhao et al. 2020).

One of the most unexpected findings of this study was the increased biomass accumulation observed in both single and double mutant plants. This result contrasts sharply with previous observations in a *dic2-1* mutant, which exhibit reduced vegetative growth that cannot be rescued by *AtDIC1* or *AtDIC3* (Lee et al. 2021). Intriguingly, photosynthetic performance remained largely unaffected in *dic2-1* plants, whereas in the present study, enhanced biomass accumulation was accompanied by increased photosynthetic efficiency and elevated chlorophyll re-oxidation capacity, providing strong evidence for improved photosynthetic performance in single and double mutants. The observed accumulation of photosynthetic pigments in the mutants is likely to support their higher photosynthetic capacity. Interestingly, discrepant phenotypes have also been reported for independent *DIC2* mutant lines. Unlike the phenotype described by Lee et al. (2021), Arabidopsis *dic2* knockout lines generated using CRISPR/Cas9 did not display any detectable developmental defects under standard growth conditions (Lv et al. 2026). Together, these observations suggest that the phenotypic manifestation of DIC2 dysfunction may be strongly influenced by genetic background, growth conditions, or the nature of the mutation itself.

Impaired growth is generally considered a hallmark of mitochondrial dysfunction in plants, particularly in mutants affecting the composition or assembly of electron transport chain complexes (Schwarzländer and Finkemeier 2013). For example, plants lacking the respiratory chain component cytochrome c (CYTc) display delayed germination, altered developmental progression, and ultimately reduced seed yield (Racca et al. 2018). These phenotypes are associated with metabolic imbalances, including daytime accumulation of starch and glucose, and are further modulated by a pronounced crosstalk between respiratory activity and gibberellin signaling. In contrast, the enhanced biomass accumulation observed in the *atdic* mutants suggests that perturbations in mitochondrial metabolite transport may elicit distinct compensatory metabolic and signaling responses, leading to developmental outcomes that differ substantially from those typically associated with respiratory chain dysfunction. Supporting this interpretation, the *atdic* mutants failed to induce *AOX1a*, a classical marker of mitochondrial stress and respiratory perturbation (Maxwell et al. 1999; Vanlerberghe et al. 2013). Furthermore, *dic2-1* mitochondria showed no significant alterations in ETC activity, TCA cycle enzymes, or enzyme kinetics, indicating that the mutant phenotype does not stem from respiratory or TCA cycle defects, but rather by altered mitochondrial metabolite exchange resulting from *DIC2* loss, potentially accompanied by compensatory metabolic adjustments (Lee et al. 2021). In this context, the increased respiratory O₂ consumption observed in *atdic3* and *atdic1/atdic3* indicates enhanced respiratory flux and was accompanied by a lower NADH/NAD⁺ ratio, consistent with a more oxidized cellular redox state. In *atdic3*, this increased respiratory flux was also accompanied by higher ATP levels, suggesting a greater contribution of mitochondrial respiration to cellular energy supply. In contrast, despite displaying the highest respiratory activity, the double mutant did not accumulate significantly more ATP, indicating that increased respiratory flux does not necessarily translate into higher ATP accumulation. Together, these results suggest that loss of DIC function alters mitochondrial metabolic flux and redox balance, with genotype-dependent consequences for cellular energy homeostasis.

Remarkably, the observed increase in shoot biomass was not paralleled by enhanced primary root elongation, a phenotype reminiscent of loss-of-function mutants affecting the E1 and E2 subunits of the mitochondrial 2-oxoglutarate dehydrogenase complex (2-OGDH; Condori-Apfata et al. 2019; 2021). A plausible explanation is that this increase in shoot biomass reflects a reallocation of carbon and energy toward shoot growth, rather than a uniform stimulation of whole-plant growth. In this context, all *atdic* mutant lines displayed an intriguing shift in carbon partitioning in the middle of the light period, characterized by reduced sucrose levels and increased starch accumulation. Conversely, the 2-OGDH mutant plants accumulated both sugars simultaneously at this stage of the photoperiod, likely reflecting a global reduction in the demand for carbon skeletons. Collectively, these observations suggest that alterations in mitochondrial metabolism can substantially impact photoassimilate utilization and carbon allocation, potentially through altered demand for carbon skeletons or changes in cytosolic redox status. In our case, the accumulation of starch despite enhanced photosynthesis further supports the idea that downstream carbon utilization is constrained, favoring storage over transport. Such disturbances in carbon allocation likely contribute to the observed phenotypes, including enhanced vegetative growth, early flowering, and reduced seed yield.

As mentioned, redox analyses further demonstrated that *AtDIC3* disruption, but not *AtDIC1*, results in a significant reduction in the NADH/NAD⁺ ratio accompanied by an increase in the NADPH/NADP⁺ ratio, identifying AtDIC3 as an important regulator of cellular redox balance. Consistent with this role, metabolomic profiling revealed preferential accumulation of amino acids, including serine, alanine, and proline, in the *atdic3* mutant, together with specific alterations in arginine and proline metabolism. Proline accumulation is particularly noteworthy given its established function in redox buffering and stress tolerance (Szabados and Savouré 2010). These metabolic adjustments may contribute to the enhanced photosynthetic efficiency and altered stress responses observed in *atdic3* plants. In contrast, the *atdic1* mutant exhibited stronger perturbations in sugar metabolism and ascorbate-related pathways, suggesting distinct contributions of AtDIC1 and AtDIC3 to the coordination of carbon and redox metabolism. In agreement with our findings, ascorbate accumulation has been reported in tomato plants with reduced mitochondrial malate dehydrogenase activity, which also display increased aerial biomass, early flowering, and enhanced photosynthetic performance (Nunes-Nesi et al. 2005). Ascorbate plays a central role in regulating photosynthetic redox balance through its involvement in the Mehler–peroxidase reaction and associated antioxidant systems (Smirnoff and Wheeler 2000). Interestingly, Lee et al. (2021) reported that disruption of *DIC2* results in increased nighttime sucrose consumption and enhanced TCA cycle–driven respiration as a compensatory response to disrupted organic acid oxidation. Such adjustments align with the established role of malate and citrate shuttles in maintaining cellular redox balance in photosynthetic tissues (Igamberdiev 2020; Zhao et al. 2020).

Our data further revealed a strong transcriptional induction of *AtDIC2* in both single and double mutants, suggesting the activation of compensatory mechanisms. Although the functional consequences of this upregulation remain unclear, it may contribute to the enhanced biomass phenotype, potentially through an increased transport capacity. This compensatory response may also explain the relatively limited number of metabolites showing differential accumulation between mutants and WT plants, as AtDIC2 activity could partially mask metabolic alterations arising from the loss of AtDIC1 or AtDIC3. Within this context, although we did not detect significant changes in the expression of other predicted dicarboxylate transporters, additional compensatory mechanisms, as proposed by Lee et al. (2021), may still contribute to buffering metabolic perturbations. However, the persistence of specific phenotypes in the mutants indicates that AtDIC2 cannot fully compensate for the functions of the other isoforms, particularly during seed germination and in the regulation of cellular redox homeostasis. Together, these observations support the notion that individual DICs function within partially distinct metabolic contexts. Consistent with this interpretation, Lv et al. (2026) reported that the overexpression of an apple DIC2 homolog in both *A. thaliana* and Micro-Tom tomato enhanced salt tolerance without causing detectable growth phenotypes under standard growth conditions. These findings support the idea that elevated DIC2 expression alone appears insufficient to trigger major developmental shifts in the absence of environmental stress.

Together, our results suggest that AtDIC1 predominantly contributes to early heterotrophic growth and carbon mobilization, whereas AtDIC3 appears to be involved in maintaining mitochondrial redox balance and amino acid metabolism during vegetative development. The partial loss of either function triggers compensatory enhancement of photosynthesis and metabolic reprogramming, leading to increased biomass and modest reduction in reproductive performance. These findings are consistent with the involvement of DICs in process linking carbon flux, redox homeostasis, and plant developmental transitions. The absence of a clear additive phenotype in the double mutant suggests that AtDIC1 and AtDIC3 operate within overlapping or interconnected metabolic pathways. In this context, compensatory metabolic adjustments may partially buffer the combined loss of function, potentially involving altered carbon fluxes through the TCA cycle and associated metabolic shuttles, thereby contributing to the maintenance of overall metabolic balance. Despite these insights, further work will be required to assess whether the transcriptional upregulation of *AtDIC2* is accompanied by changes at the protein and transport activity levels, and whether this potential compensatory response contributes to the physiological phenotypes observed in the *atdic1* and *atdic3* mutants.

## Supplementary Information

Below is the link to the electronic supplementary material.

Supplementary file1 (XLSX 15 KB)

Supplementary file2 (PDF 972 KB)

## Data Availability

The datasets generated during and/or analyzed during the current study are available from the corresponding author on reasonable request.
