# Supplementary material for: Knockout of mitochondrial dicarboxylate carriers AtDIC1 and AtDIC3 affects germination, biomass accumulation, and carbon partitioning in Arabidopsis thaliana
Source: Plant Cell Rep. 2026 Sep 28;45(10):309. doi: 10.1007/s00299-026-04000-7 (PMC13619700; doi:10.1007/s00299-026-04000-7)
Supplement: Supplementary file 1 [file 299_2026_4000_MOESM2_ESM.pdf]

Supplementary Table S1. List of primers used in this study.

Primer Name	Nucleotide Sequence (5' - 3')
Salk_ LB1.3	ATTTTGCCGATTTCGGAA
atdic1_Salk090789 F	ACAAAGAAAGCATCCCATGTG
atdic1_Salk090789 R	CTATTTTGCGCACAAGGAAAG
atdic1_Salk067118_F	ATGGTCGTTTACCGTTGACTG
atdic1_Salk067118_R	ATATTTTGCTGGTGGCAGTTG
atdic3_Salk001547 F	AGAACCAGAAGAGCCAGAAGG
atdic3_Salk001547 R	GGGTAATAAGGAAATGCTGCC
atdic3_Salk087822_F	TTATTTTGGGCCATGTCTCAG
atdic3_Salk087822_R	CCGTCGCAGAAACTACAAGAG
qAtDIC1_F	CGACCCACCCGCTTGAT
qAtDIC1_R	GCTGGTCGGAGATTGGTTTG
qAtDIC2_F	TCGTTGAAGGTGGGATTGC
qAtDIC2_R	CTTGATTAGATCGAGAGGGTGAGT
qAtDIC3_F	AATCTTCCCGTGAAACCTTACC
qAtDIC3_R	AAGGAAATGCTGCCGATGAGA
qAtSFC1_F	CGGCGCTTACAAAGGAATCG
qAtSFC1_R	ATCCCATCCGAAGCGTGTAC
qAtDTC_F	ATGTGGTCTGACAGCTGGTG
qAtDTC_R	AAGCGCATGGAAAGCATTGG
qAtGAPDH_F	GCCAAGGCTGGGATTGC
qAtGAPDH_R	GTCGTACCATGACACCAACTTCA
qAt40S_F	CGACTCTCTGCGTTAGGTTTCA
qAt40S_R	GGGTTTCTCCTTGCTTTTGCT
qAtAox1A_F	CCGATTTGTTCTTCCAGAGG
qAtAox1A_R	GCGCTCTCTCGTACCATTTC
qAtCOX15_F	AGTGGTTTAGAGGAGCCACCG
qAtCOX15_R	TCAGCTGGTGGTTCAGGCAT
qAtVDAC1_F	TGAGGTCAATCTTCAGCTTCAA
qAtVDAC1_R	TTTCCAGACTTGGTGTCAAATG

Supplementary Table S3. Comparison of growth-related traits and 100-seed weight between Col-0, single and double *atdic* mutant lines. Values represent mean ± SD. Different letters indicate statistically significant differences among genotypes according to Tukey's post hoc test ($p < 0.05$).

**Genotype**	**Rosette Area (cm²)**	**Fresh Weight (g)**	**Dry Weight (g)**	**Seed Weight (mg)**
Col-0	0.31 ± 0.081 a	0.20 ± 0.072 a	0.04 ± 0.012 a	1.79 ± 0.168 a
atdic1	0.42 ± 0.116 b	0.30 ± 0.061 b	0.06 ± 0.012 b	1.81 ± 0.248 a
atdic3	0.45 ± 0.117 b	0.27 ± 0.036 b	0.06 ± 0.005 b	2.10 ± 0.173 b
atdic1/atdic3	0.49 ± 0.081 b	0.30 ± 0.058 b	0.06 ± 0.014 b	1.81 ± 0.219 a

Supplementary Table S4. Chlorophyll *a* fluorescence parameters in the *atdic* mutants under increasing light intensities. [#]

**Genotype**	ϕ_{PSII}	***ETR***	***qP***	***NPQ***	***Fv'/Fm'***	***D***	***Ex***
**WT COL-0**	0.23	46.7	0.351	0.93	0.646	0.354	0.416
atdic1	0.260 *	54.8 *	0.399 *	0.95	0.642	0.358	0.382
atdic3	0.254 *	51.9 *	0.371	0.88	0.676 *	0.324 *	0.422
atdic1/atdic3	0.266 *	54.5 *	0.387	0.80 *	0.679 *	0.321 *	0.413
**PPFD µmol m^{-2} s^{-1}**	***ϕPSII***	***ETR***	***qP***	***NPQ***	***Fv'/Fm'***	***D***	***Ex***
**125 (control)**	0.348	18.2	0.511	0.64	0.681	0.319	0.334
**190**	0.393 *	31.4 *	0.563	0.55	0.698	0.302	0.305
**285**	0.336	40.3 *	0.48	0.48 *	0.704	0.296	0.368
**420**	0.257 *	45.3 *	0.375 *	0.67	0.686	0.314	0.430 *
**625**	0.209 *	54.9 *	0.315 *	0.94 *	0.664	0.336	0.454 *
**845**	0.190 *	67.5 *	0.295 *	1.12 *	0.646 *	0.354 *	0.456 *
**1150**	0.152 *	73.5 *	0.250 *	1.28 *	0.609 *	0.391 *	0.457 *
**1500**	0.135 *	85.0 *	0.227 *	1.42 *	0.597 *	0.403 *	0.462 *
**Statistics**	***ϕPSII***	***ETR***	***qP***	***NPQ***	***Fv'/Fm'***	***D***	***Ex***
**Genotype**	P<0.05	P<0.01	P<0.05	P<0.01	P<0.001	P<0.001	P<0.05
**PPFD**	P<0.001	P<0.001	P<0.001	P<0.001	P<0.001	P<0.001	P<0.001
**Genotype x PPFD**	NS[†]	NS	NS	NS	NS	NS	NS

[#] Multiple comparisons were performed using the Holm–Šidák method, with the control group as reference. [†] NS – NOT SIGNIFICANT

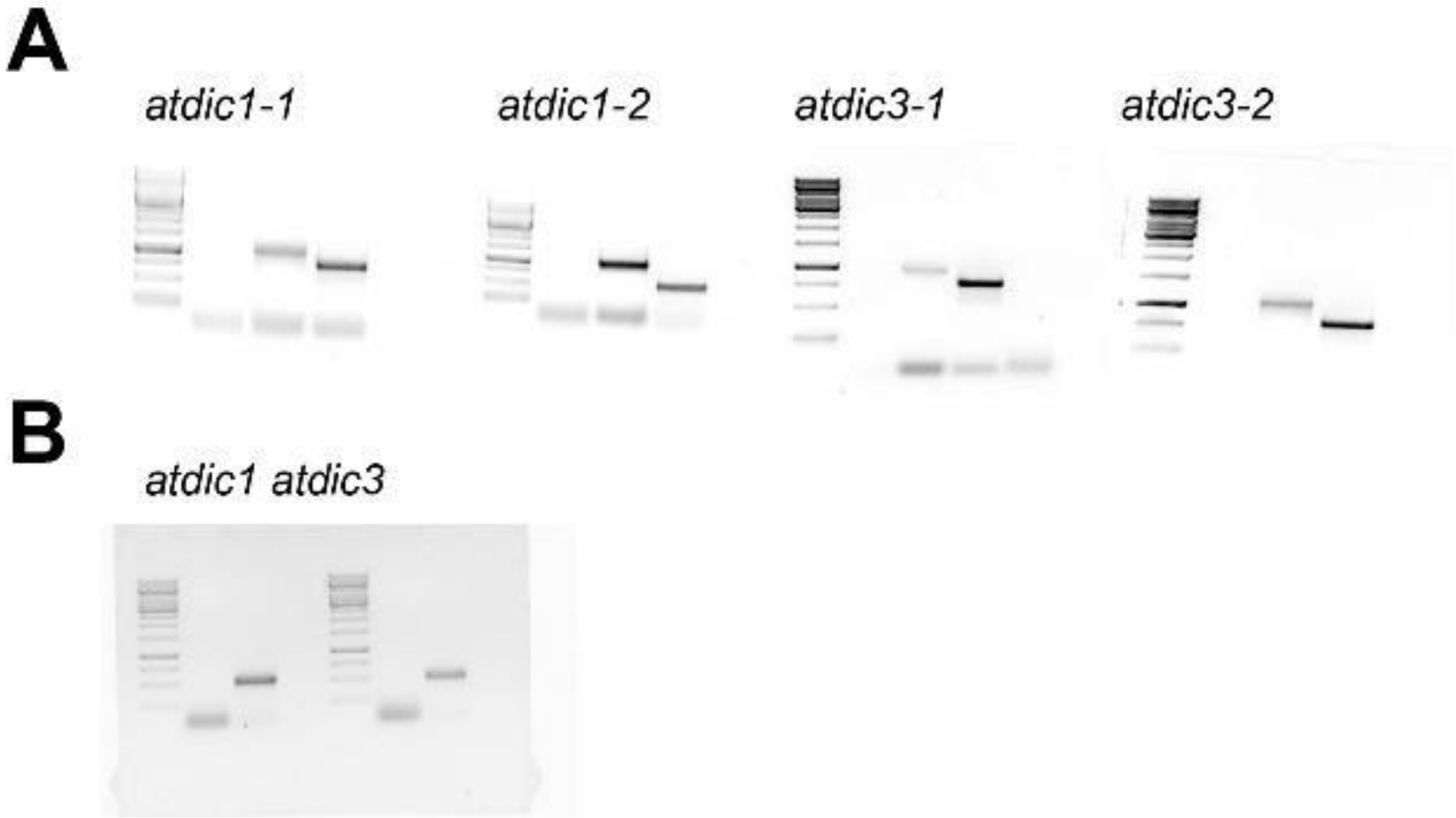

Supplementary Fig. S1. Confirmation of homozygous single and double T-DNA insertion mutants by genomic PCR. (A) *atdic1* and *atdic3* single mutant lines. (B) *atdic1/atdic3* double mutant generated from a cross between *atdic1-1* and *atdic3-1*. PCR products for *AtDIC1* and *AtDIC3* were generated using gene-specific primers and a left border primer (Supplementary Table S1), following the protocol described in Methods. The PCR products were separated on a 0.8% agarose gel and visualized with ethidium bromide staining.

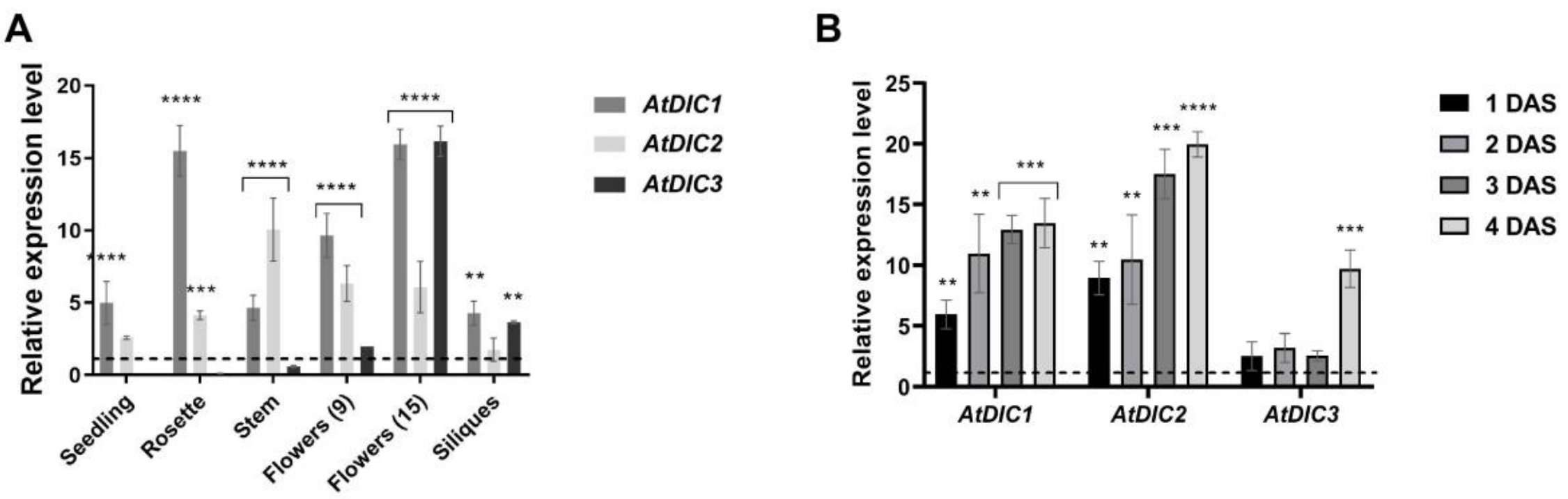

Supplementary Fig. S2. Expression patterns of *AtDIC1, AtDIC2* and *AtDIC3* in various Arabidopsis organs/tissues and in germinating seeds. Relative expression was determined by RT-qPCR in (A) 14-day-old seedlings, vegetative rosette leaves (growth stage 3.5; Boyes et al. 2001), stems, flowers at stages 9 and 15 (as classified by Smyth et al. 1990), and siliques; and (B) germinating seeds (1 to 4 days after stratification). A pooled sample of these organs/tissues was used as a calibrator (set to 1; dashed line). Asterisks indicate significant differences from WT as determined by one-way ANOVA followed by Tukey's test (**p-value<0.01, ***p-value<0.001 and ****p-value<0.0001).

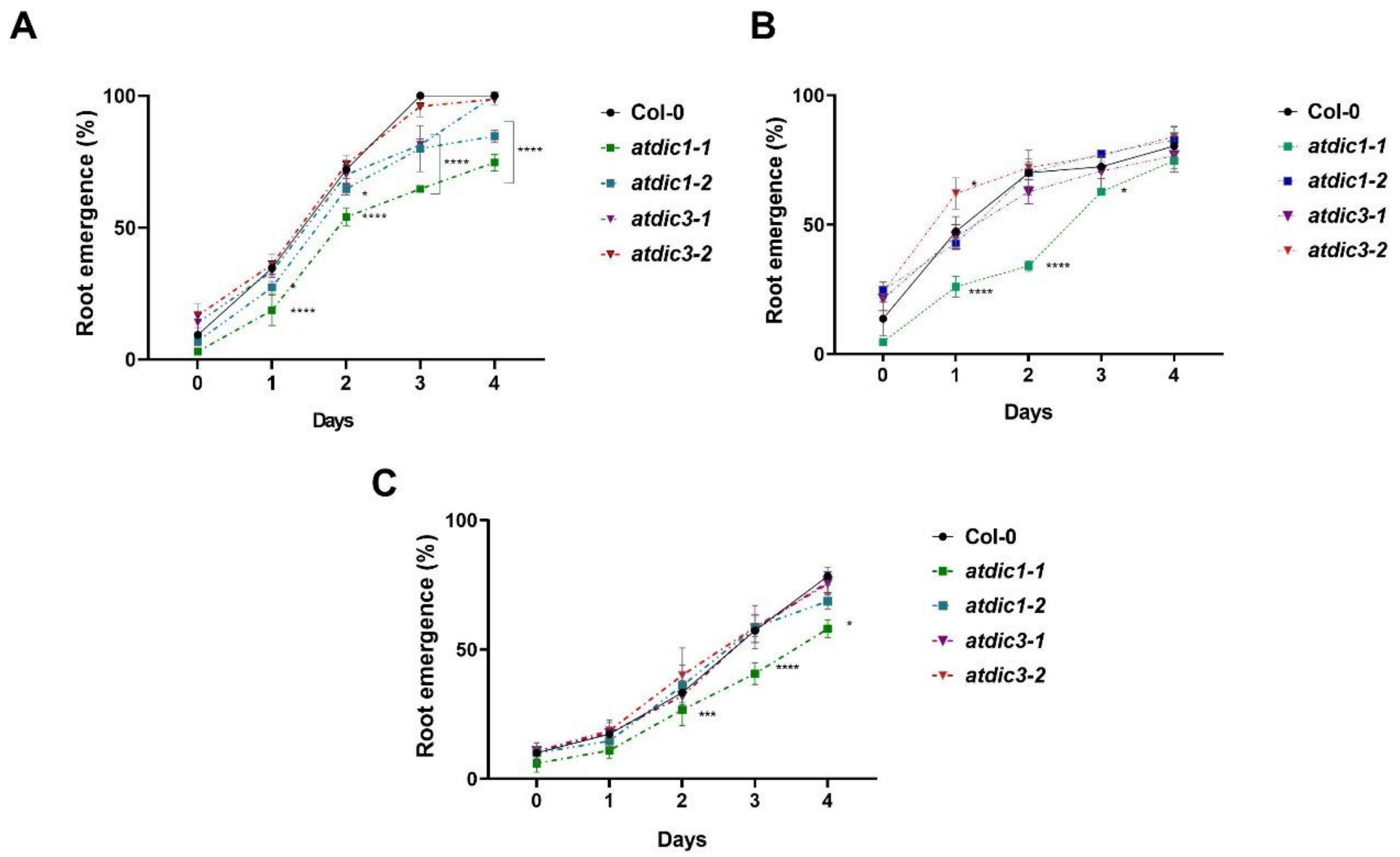

Supplementary Fig. S3. Germination rates (%) of wild type (Col-0) and single mutant seeds plated on half-strength MS medium without sucrose. (A) control condition; (B) medium supplemented with 200 mM mannitol; (C) medium supplemented with 100 mM NaCl. Data represents the means of three independent assays, each using 30 seeds per genotype per plate, measured over a 4-day period. Asterisks indicate significant differences relative to the WT by two-way ANOVA with Dunnett's multiple comparison test (*p<0.05, ***p<0.001 and ****p<0.0001).

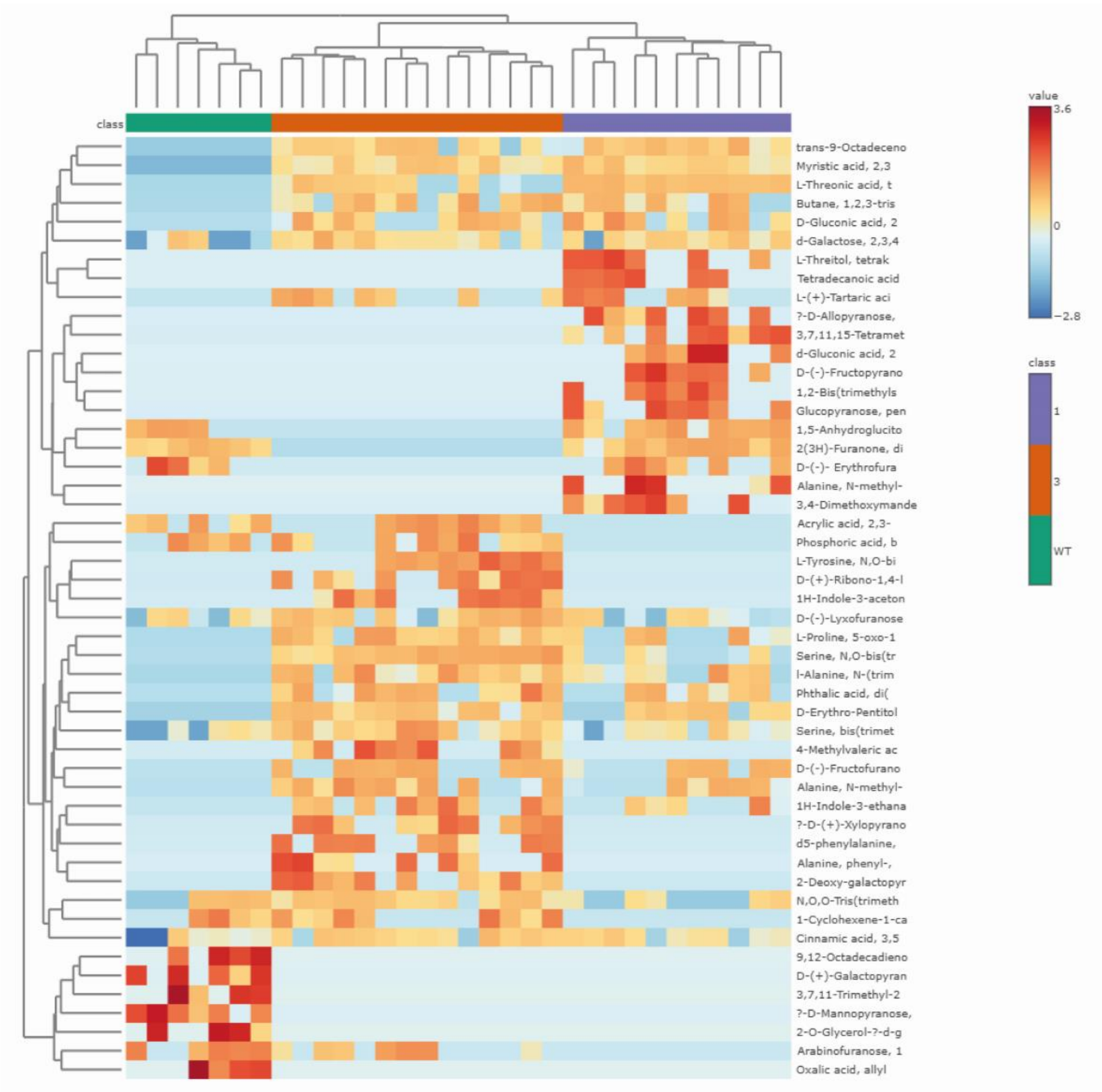

Supplementary Fig. S4. Metabolic profiles of *atdic1* and *atdic3* single insertion mutants. Heatmap representation of the 50 most significantly altered rosette (35 DAS) metabolites after normalization and data processing. Metabolite levels are shown as fold changes relative to wild type (Col-0). A blue-to-red color scale represents relative abundance, with blue indicating decreased levels and red indicating increased levels.

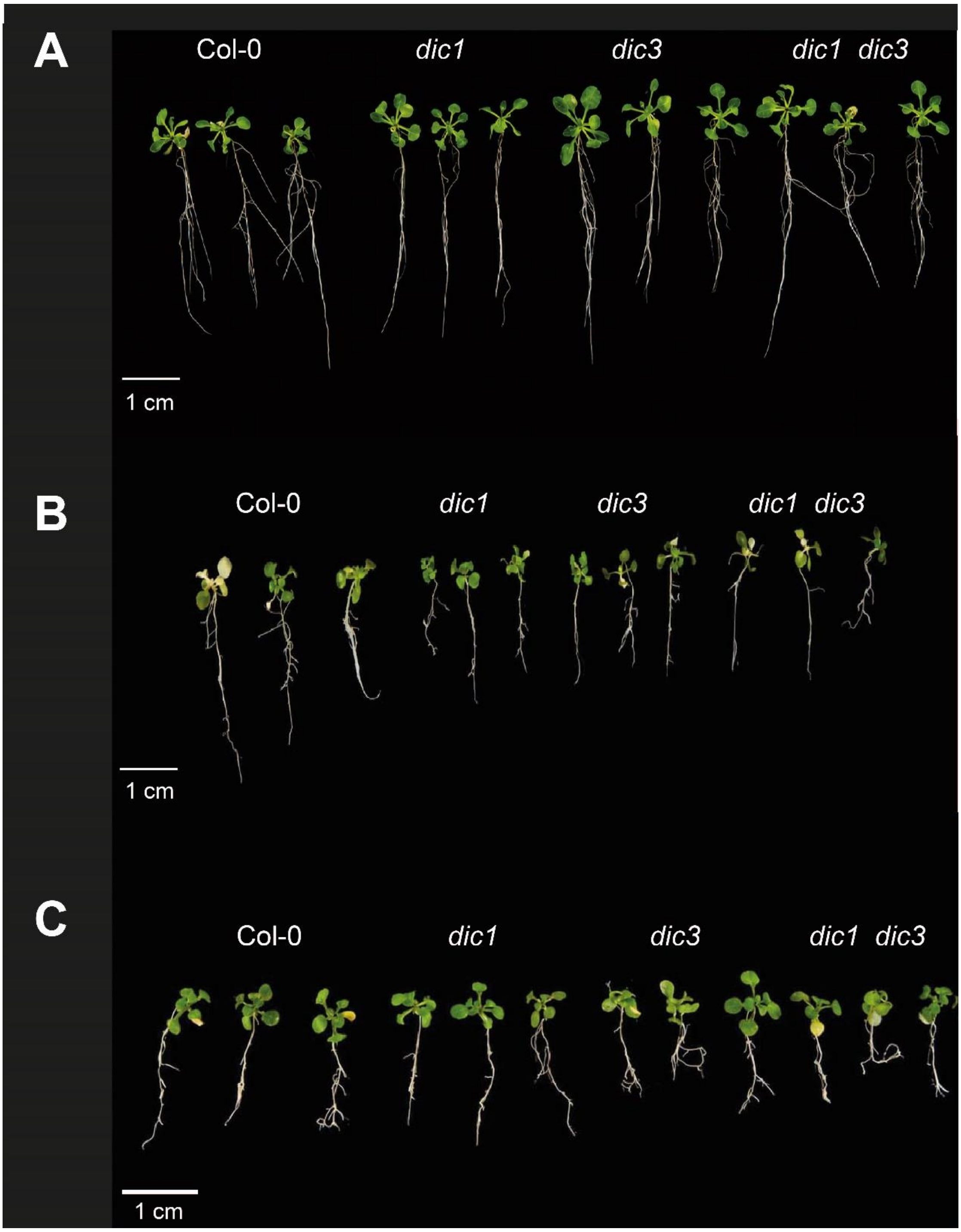

Supplementary Fig. S5. Primary root length of *atdic* mutants under control and stress conditions. Representative images of 10-day-old Col-0, *atdic1*, *atdic3*, and *atdic1/atdic3* seedlings grown under control conditions (A), NaCl-induced salt stress (B), and mannitol-induced osmotic stress (C). Primary root length was measured for each genotype under the respective conditions. Scale bars = 1 cm.

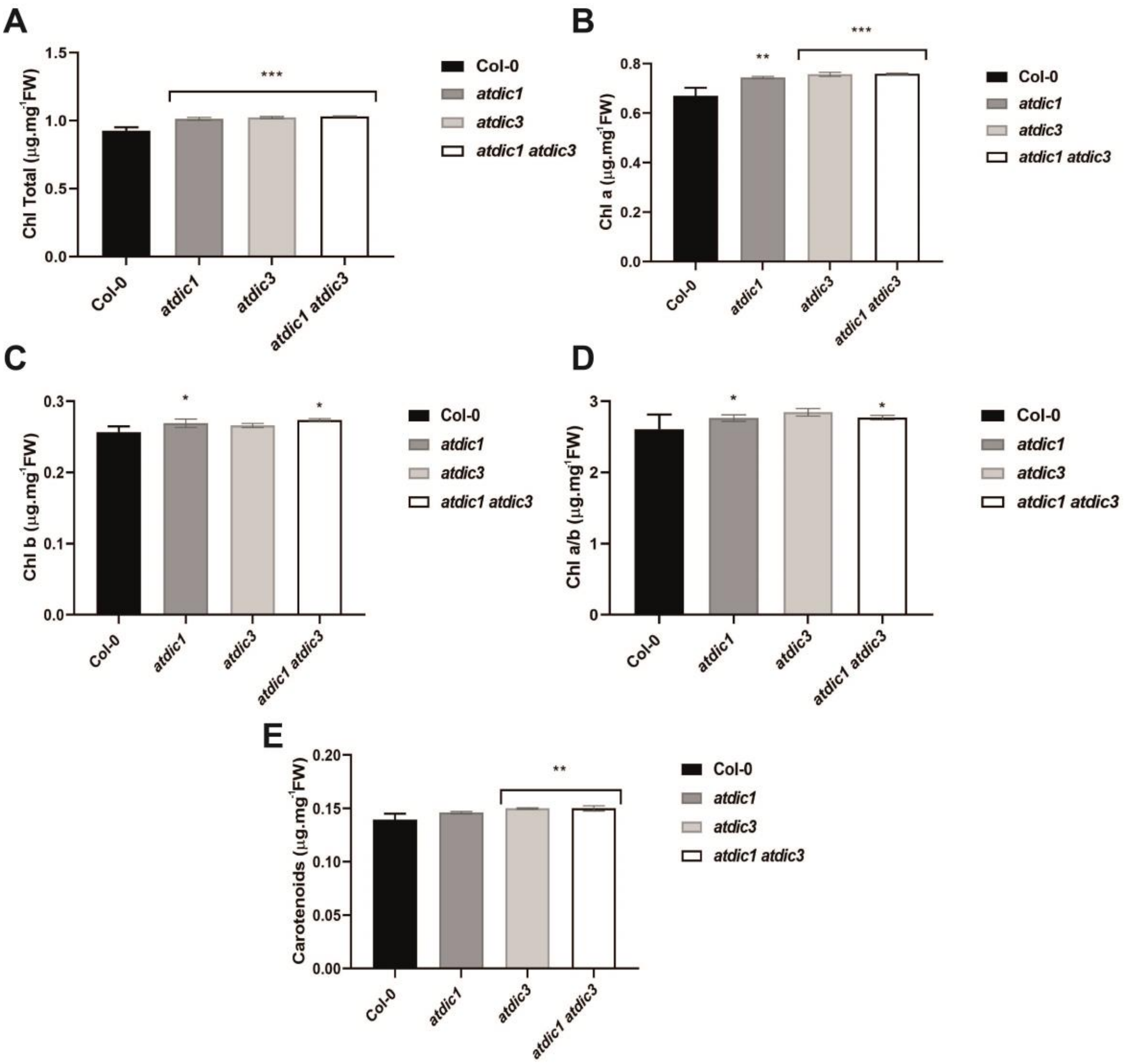

Supplementary Fig. S6. Photosynthetic pigment contents in leaves of wild type (Col-0), single, and double mutant plants (35 DAS). A) Total chlorophyll; B) chlorophyll *a*; C) chlorophyll *b;* D) chlorophyll *a/b* ratio; E) total carotenoids. Data represents the means (± SD) of three independent assays, each using detached leaves (8th and 9th) from five individual plants per genotype. Asterisks indicate significant differences from WT as determined by one-way ANOVA followed by Tukey's test (*p-value<0.05, **p-value<0.01 and ***p-value<0.001).

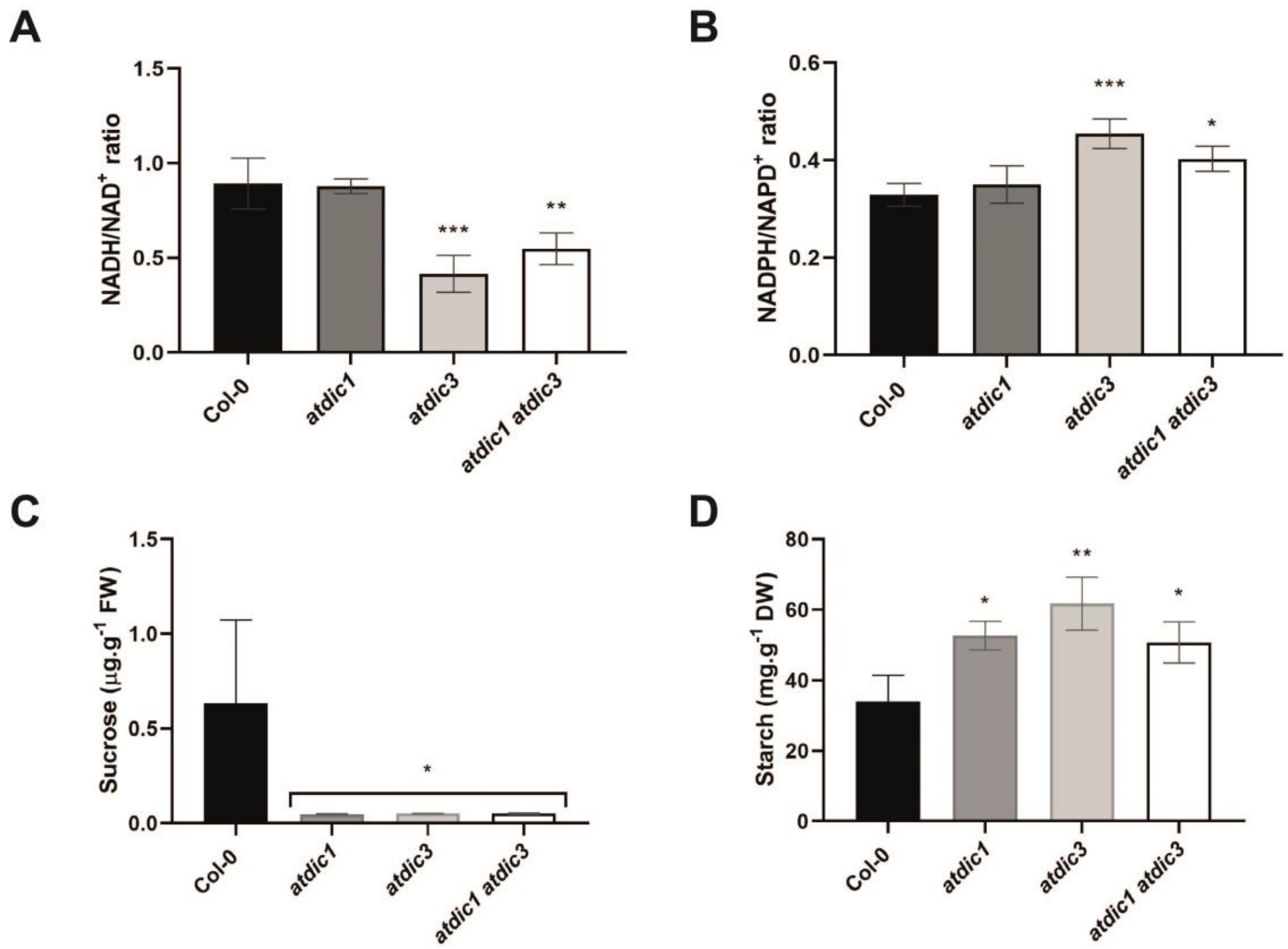

Supplementary Fig. S7. Redox status and sucrose and starch contents in wild type (Col-0), single, and double mutant plants (35 DAS). A) $NADH/NAD^+$ ratio; B) $NADPH/NADP^+$ ratio; C) sucrose; D) starch. Data represents the means (± SE) of five individual plants per genotype. Asterisks indicate significant differences from WT as determined by one-way ANOVA followed by Tukey's test (*p-value<0.05, **p-value<0.01 and ***p-value<0.001).

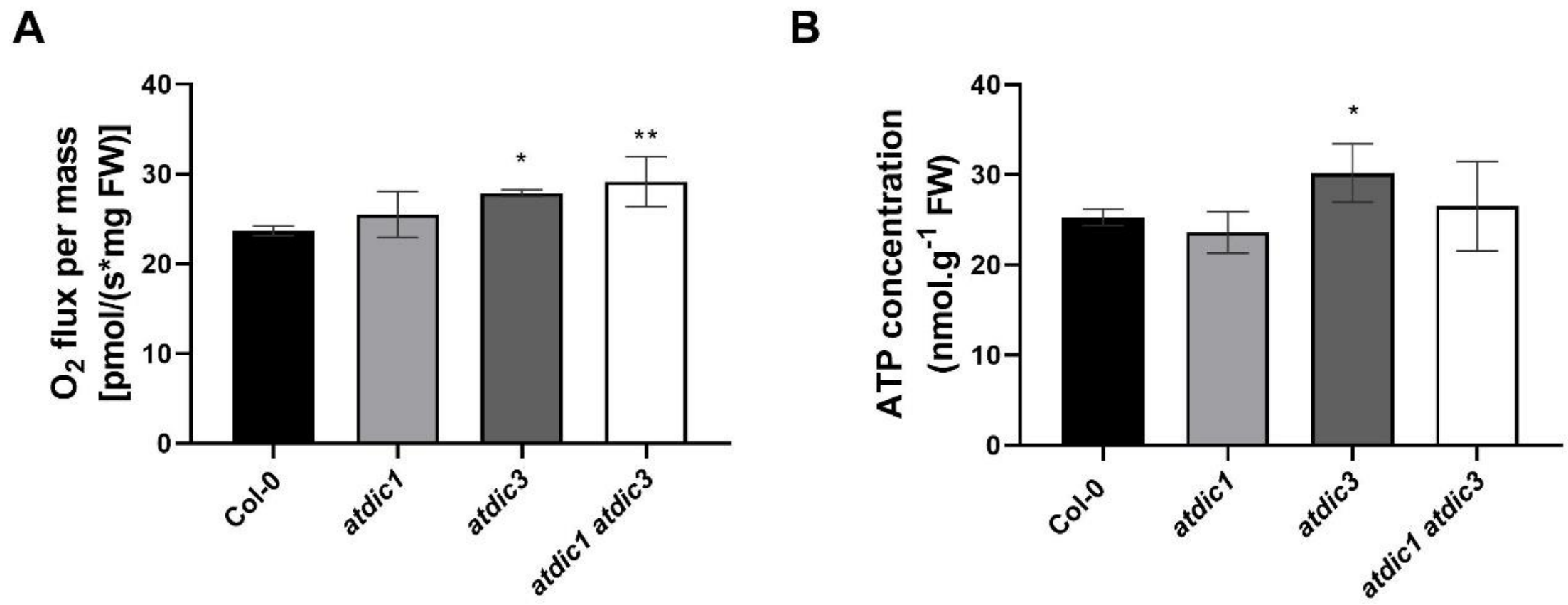

Supplementary Fig. S8. Respiratory O_2 flux and ATP content in *Arabidopsis thaliana* DIC mutants. (A) O_2 flux per unit fresh weight measured in leaves of Col-0, *atdic1*, *atdic3*, and *atdic1/atdic3* plants. (B) ATP concentration in leaves of the same genotypes. Values represent means ± SD of four independent biological replicates. Asterisks indicate significant differences from WT as determined by one-way ANOVA followed by Tukey's test (*p-<0.05, **p<0.01).
